# Seasonal Variation in the Intake of Food Groups and Nutrients in Japan: A Systematic Review and Meta-analysis

**DOI:** 10.2188/jea.JE20240139

**Published:** 2025-02-05

**Authors:** Riho Adachi, Fumi Oono, Mai Matsumoto, Xiaoyi Yuan, Kentaro Murakami, Satoshi Sasaki, Hidemi Takimoto

**Affiliations:** 1 Department of Health and Social Behavior, School of Public Health, The University of Tokyo, Tokyo, Japan; 2 Department of Social and Preventive Epidemiology, Division of Health Sciences and Nursing, Graduate School of Medicine, The University of Tokyo, Tokyo, Japan; 3 Department of Nutritional Epidemiology and Shokuiku, National Institutes of Biomedical Innovation, Health, and Nutrition, Osaka, Japan; 4 Department of Social and Preventive Epidemiology, School of Public Health, The University of Tokyo, Tokyo, Japan

**Keywords:** season, food, nutrient, variation, Japan

## Abstract

**Background:**

Seasonal variations could systematically bias dietary intakes. This systematic review aimed to determine seasonal variations in dietary intake among Japanese adults.

**Methods:**

PubMed and Ichushi-Web databases were searched for studies reporting seasonal intakes of nutrients or food groups assessed using dietary records or 24-hour recalls. The protocol was registered with PROSPERO (CRD42022356084).

**Results:**

Ten studies (eight studies on 1–31 nutrients and four on 2–15 food groups) met the inclusion criteria. Six studies included both sexes, whereas four investigated only females. The number of participants ranged from 25 to 459, and the number of dietary survey days in each season ranged from 1 to 14. For most nutrient and food groups, the reported seasonal variations were inconsistent across studies. The meta-analyses comparing differences in mean intakes between two seasons showed no significant differences in all comparisons or differences in only one comparison for most nutrients and food groups. Significant seasonal differences were observed for vegetables, fruits, and potatoes in five out of six comparisons, though the heterogeneity was high. Their biggest differences were as follows: 101 g/day more vegetable intake in summer than spring, 60 g/day more fruit intake in fall than spring, and 20.1 g/day more potato intake in fall than spring.

**Conclusion:**

Reported seasonal variations were inconsistent across studies for most food groups and nutrients. Relatively distinct seasonal differences in mean intakes were observed for vegetables, fruits, and potatoes in meta-analyses. However, these results must be interpreted cautiously because of the high heterogeneity and limited representativeness.

## INTRODUCTION

Four seasons (spring, summer, fall, and winter) are characterized by different temperatures and precipitation.^1^ The differences in weather patterns may affect food production. Consequently, food availability may differ for each season, which can alter an individual’s food choices. Several studies have examined seasonal variations of dietary intake and found no common seasonal patterns.^2^^–^^12^ A systematic review and meta-analysis was conducted using the data obtained from 21 different countries, but it synthesized the dietary data without considering regional cultural areas.^13^ Not only the seasons but also food culture varies by region and country^14^; therefore, reports on seasonal variations in dietary intake synthesized by each country or region would be helpful to design dietary surveys and interpret their results.

It remains unclear if systematic seasonal variations exist in the intake of food groups and nutrients in the Japanese population. Local cultural habits might cause seasonal variations in dietary intake.^13^ For example, the Japanese government has facilitated people to consume seasonal foods because such foods are the most nutritious throughout the year and let people appreciate seasonal transitions.^15^ In fact, nutrient content differs by season, even in the same food, such as spinach, which has three times higher vitamin C content in winter than in summer.^16^ Conversely, in industrialized countries, such as the United States, seasonal dietary intake variations are considered negligible due to improved preservation and transportation facilities.^6^^,^^17^ Food security in Japan is assumed to be satisfactory, and diverse food availability is maintained through imports all year long.^18^ Therefore, seasonal variation in dietary intake remains uncovered for the Japanese population, as the food culture and the food environment may counterbalance each other.

Although caution is advised to deal with seasonality in epidemiological studies on dietary intake,^8^^–^^11^^,^^13^ it would be practically difficult to conduct dietary surveys repeatedly to assess habitual dietary intake, especially in a nationally representative population, due to monetary and labor constraints. In Japan, the National Health and Nutrition Survey, which provides nationally representative dietary intake patterns of the Japanese population, has been collecting dietary data using only a 1-day dietary record in November.^19^^,^^20^ Hence, it is crucial to examine if there is any seasonal variation in the intake of nutrients and food groups in the Japanese population. If any seasonal variation exists, it would be recommended to reconsider the research season based on the degree of seasonal differences in dietary intake.

Therefore, this systematic review aimed to answer the following questions: (i) does seasonal variation exists in the intake of food groups and nutrients in the Japanese population? and (ii) if so, what is the degree to which the difference in the intake of foods or nutrients depends on seasons?

## METHODS

The protocol for this systematic review was registered in the International Prospective Register of Systematic Reviews (PROSPERO: CRD42022356084). This review was conducted following the Preferred Reporting Items for Systematic reviews and Meta-Analyses guidelines (PRISMA).^21^

Search strategy and eligibility criteria. We searched the electronic databases: PubMed and the Ichushi-Web (Japanese medical literature database) from January 1990 through 28 September 2022 to identify studies describing seasonal variation in the intake of food groups and nutrients in Japan. The search terms entered into PubMed were ‘(periodicity OR season OR seasonal OR seasonality OR (spring AND summer AND (fall OR autumn) AND winter)) AND (diet OR dietary OR consum^*^ OR intake^*^ OR eating) AND (Japan OR Japanese) AND (("dietary record" OR "dietary records" OR "diet record" OR "diet records" OR "food diary" OR "food diaries" OR "food record" OR "food records") OR (recall OR recalls))’. The search terms entered into the Ichushi-Web were the Japanese words for ‘(season OR (spring AND summer AND ("fall OR autumn") AND winter)) AND intake’ and the search condition was restricted to original papers.

The inclusion criteria for the studies were as follows: (i) the mean or median dietary intake value of Japanese individuals was reported for two or more out of four seasons; (ii) more than 90% of the same individuals were examined over seasons; (iii) dietary records or dietary recalls were adopted as the dietary survey method; (iv) the same dietary survey method was employed throughout the study period; (v) intake of at least one nutrient or food group reported in the Japan National Health and Nutrition Survey was examined^22^; and (vi) research conducted in free-living settings. The exclusion criteria were as follows: (i) research that exclusively included patients, people with high physical activity level, pregnant women, lactating women, or infants and children; (ii) research conducted in experimental conditions; or (iii) papers written in languages other than English and Japanese. Two authors (RA and FO) independently reviewed the records identified in each database. The title and abstract of each identified article were checked, followed by full-text screening. All disagreements were discussed until a consensus was reached.

The quality of the included studies was assessed using the Appraisal tool for Cross-Sectional Studies (AXIS),^23^ which was modified to fit the purpose of this study. We adopted 16 of the 20 components from the AXIS for the Introduction, Methods, and Results sections. We did not use four components on Discussion and Other in the AXIS because we focused on the methods and results of each study. Moreover, we considered the studies to have a low risk of bias if the number of survey days was multiplied by the number of participants over 150, because this enabled us to detect an approximately 30% difference in the standard deviation under a power of 0.8 and a significance level of 0.05. These values were decided arbitrarily by the authors’ knowledge to identify a meaningful difference in dietary intake. Low-risk-of-bias studies were awarded one point, and high-risk-of-bias studies were awarded 0 points for each component. The points awarded ranged from 0 to 17. Two authors (RA and FO) independently assessed the quality, and all disagreements were discussed until an agreement was obtained. The detailed criteria for quality assessment are described in eTable 1.

The following general characteristics were extracted from each article: first author; journal; publication year; geographical area where the research was conducted; survey year; participant characteristics including age and number of participants; dietary assessment method; number of survey days; definition of seasons (months); and season at which the survey was started. Furthermore, we extracted data on the mean or median intakes of energy, nutrients, food groups, and whether (or not) the analysis was adjusted for energy (see eTable 2 and eTable 3). Thereafter, data on any existing significant differences in the intake of nutrients and food groups among the seasons were extracted. The intake of beverages and confectioneries was not extracted because their definitions differed across studies,^2^^,^^24^^–^^26^ which made it impossible to synthesize the data accurately. If the values of both crude intake and energy-adjusted intake by the density model were reported in the studies, the value of the former was extracted. All data were extracted by RA and double-checked by FO. Finally, we conducted meta-analyses using a random-effects model to calculate summary differences and 95% confidence intervals (CIs) in mean intakes of nutrients and food groups between two seasons and produced forest plots when a minimum of two studies reported the same outcomes. An individual’s dietary intakes between seasons were assumed to be correlated with each other with a correlation coefficient of 0.6, based on a previous study conducted in the United States reporting that dietary intakes assessed twice with 2-to-4-month intervals had correlations of around 0.4 to 0.7,^27^ as we did not have crude data from each study or available reports on the seasonal correlation of dietary intake in Japan. Sensitivity analyses were also performed assuming that dietary intakes in two seasons had no correlation (independent) for nutrients and food groups that had significant differences between two seasons. The *I*^2^ index was used to assess heterogeneity between studies. *I*^2^ values of <25%, 25–50%, and >50% were considered as low, moderate, and high heterogeneity, respectively.^28^ All statistical analyses were performed using R software (version 4.3.2; R Foundation for Statistical Computing, Vienna, Austria), and a *P*-value of 0.05 or less was considered statistically significant.

## RESULTS

**Figure 1.**
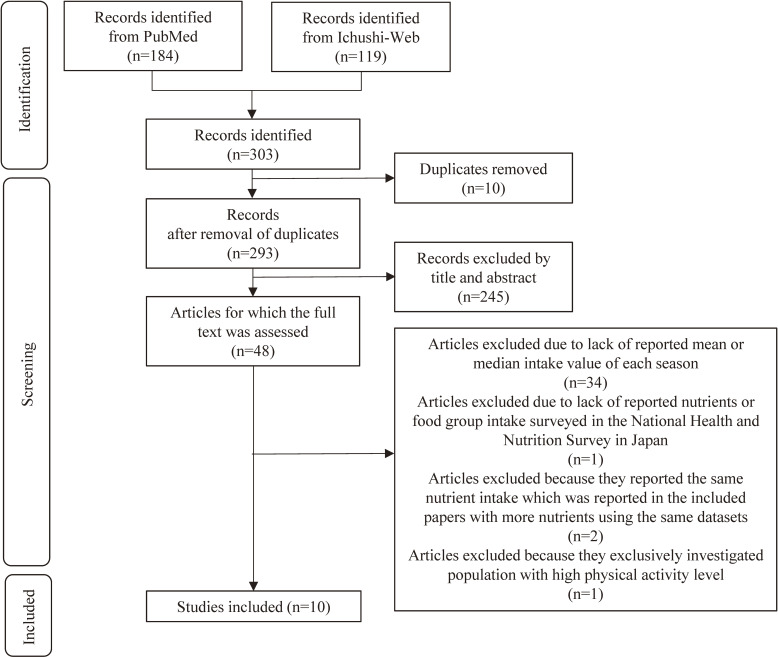
Flowchart of the study selection process

In total, 184 records were identified from PubMed, while 119 were derived from the Ichushi-Web. Ten duplicates were removed. After eliminating 245 irrelevant records through title and abstract screening, 48 full-text articles were assessed through full-text check. Finally, this review included 10 eligible studies.^2^^–^^4^^,^^24^^–^^26^^,^^29^^–^^32^ A detailed flowchart of the study selection process is depicted in Figure 1. The 10 papers were subjected to quality assessment, which yielded scores of 9 to 14 out of a maximum of 17 points (eTable 1). No apparent low-quality study allowed us to further data synthesis.

The general characteristics of the studies are summarized in Table 1, and the dietary assessment methods are summarized in Table 2. The earliest study was published in 1996, which analyzed data obtained between 1992 and 1993.^4^ Five studies conducted dietary surveys before 2000^2^^–^^4^^,^^26^^,^^31^ and the most recently published study used data acquired between 2013 and 2014.^32^ The research area covered 21 of 47 prefectures in Japan, ranging from the northernmost prefecture, Hokkaido,^25^ to the southernmost, Okinawa.^26^ Six studies investigated general populations comprising both males and females.^2^^,^^4^^,^^24^^,^^26^^,^^30^^,^^32^ In contrast, four studies were conducted exclusively among females: the general population,^25^ female dietitians,^3^ female university students,^29^ and housewives.^31^ The number of participants ranged from 25^25^ to 459.^30^ Nine of the included studies adopted the dietary record method^2^^,^^3^^,^^24^^–^^26^^,^^29^^–^^32^ for 2^24^^,^^25^ to 14^31^ days in each season, and the remaining study adopted the 24-hour recall method for 1 day in each season.^4^ Eight of the nine studies reported the following definitions of seasons: March to June as spring, July to September as summer, October to December as fall, and December to March as winter.^2^^–^^4^^,^^24^^–^^26^^,^^29^^,^^30^ Five studies reported the season for the commencement of research as fall or winter,^2^^–^^4^^,^^31^^,^^32^ one study reported as spring,^29^ one study reported as summer,^24^ and the season was unknown for the remaining studies.^25^^,^^26^^,^^30^

**Table 1.** General characteristics of each study

1^st^ Author	Publication year	Survey year	Prefectures	Municipalities	Participants	Age, years	Number of participants (completed all seasons)
Owaki (4)	1996	1992–1993	Gifu	N/A	General people living in the research area	35 or over	143
Tokudome (3)	2002	1996–1997	Aichi	N/A	Female dietitians	32–66	80
Sasaki (2)	2003	1994^a^	Iwate, Akita, and Nagano	Ninohe, Yokote, Saku	General people living in the research area	44–63^a^	160
Amano (29)	2007	2001–2003	Kinki area	N/A	Female university students	mean 19.2	85
Ishiwaki (30)	2007	2004–2005	12 prefectures (Aomori, Akita, Iwate, Yamagata, Nagano, Gunma, Chiba, Okayama, Tokushima, Kochi, Fukuoka, and Miyazaki)	N/A	General people living in the research area	50–69	459
Nozue (24)	2008	2003–2004	Toyama	N/A	Volunteers for Promoting Improved Dietary Habits and their family	20 or over	90
Miyai (25)	2011	2007–2009	Hokkaido	Bifuka Town	Females living in the research area	30 or over	25
Tatsumi (26)	2014	1996–1998	Niigata, Ibaraki, Osaka, Kochi, Nagasaki, Okinawa	Kashiwazaki, Mito, Suita, Chuo-higashi, Kamigoto, Miyako	General people living in the research area	39–77	390
Minari (31)	2016	1999–2000	Fukuoka	Shime Town	Housewives living in the research area	55–65	28 (27)
Akimoto (32)	2019	2013–2014	Shizuoka	Shizuoka City	General people living in the research area	mean 43.5	78 (51)

^a^This information was extracted from the cohort profile paper published in the same issue. (Tsugane S, Sasaki S, Kobayashi M, et al. Validity and reproducibility of the self-administered food frequency questionnaire in the JPHC Study Cohort I: study design, conduct and participant profiles. *J Epidemiol.* 2003 Jan;13:S2–S12).

**Table 2.** Dietary assessment method of each study

1^st^ Author	Dietary assessment method	Number of survey days in each season	Definition of seasons by month				The season for the commencement of research

			Spring	Summer	Fall	Winter	
Owaki (4)	24-hour recall	1	4	7	10	1	Fall
Tokudome (3)	Weighed dietary record	7	4	7–8	10–11	1	Fall
Sasaki (2)	Weighed dietary record	7	5–6^a^	8–9^a^	11–12^a^	2–3^a^	Winter^a^
Amano (29)	Weighed dietary record with pictures	3	4	7	10	1	Spring
Ishiwaki (30)	Weighed dietary record	3	5–6	8–9	11–12	2–3	N/A
Nozue (24)	Weighed dietary record	2	3	8	12	N/A	Summer
Miyai (25)	Weighed dietary record	2	4–6	7–9	10–11	12–3	N/A
Tatsumi (26)	Weighed dietary record	7	5	8	11	2	N/A
Minari (31)	Weighed dietary record	14	N/A	N/A	N/A	N/A	Fall^b^
Akimoto (32)	Weighed dietary record	3	N/A	N/A	N/A	N/A	Winter

^a^This information was extracted from the cohort profile paper published in the same issue. (Tsugane S, Sasaki S, Kobayashi M, et al. Validity and reproducibility of the self-administered food frequency questionnaire in the JPHC Study Cohort I: study design, conduct and participant profiles. *J Epidemiol.* 2003 Jan;13:S2–S12).

^b^The study started in November, and we interpreted it as fall.

### Reported seasonal differences

Table 3 presents the reported differences in the intake of energy and nutrients among seasons. Nine^2^^–^^4^^,^^24^^,^^25^^,^^29^^–^^32^ of the 10 included studies investigated 1 to 31 nutrients. Nine studies investigated the intake of energy,^2^^–^^4^^,^^24^^,^^25^^,^^29^^–^^32^ and eight studies investigated the intake of protein^2^^–^^4^^,^^24^^,^^29^^–^^32^ and fat.^2^^–^^4^^,^^24^^,^^29^^–^^32^ On the contrary, only one study reported the intake of n-6 polyunsaturated fatty acid (PUFA)^3^ and n-3 PUFA.^3^ The proportion of nutrients with significant differences varied across studies. Two studies reported significant differences for more than 80% of the nutrients examined,^3^^,^^31^ while the other studies showed differences in 0 to 40% of nutrients examined.^2^^,^^4^^,^^24^^,^^25^^,^^29^^,^^30^^,^^32^ Vitamin C intake was investigated in seven studies,^2^^–^^4^^,^^24^^,^^29^^–^^31^ most of which reported significant differences across seasons. Table 4 presents the reported differences in the intake of food groups among the four seasons. Four of ten studies investigated 2 to 15 food groups.^2^^,^^24^^–^^26^ Three studies investigated vegetables and fruits,^2^^,^^24^^,^^26^ and all of which reported significant differences across studies for both male and females.

**Table 3.** Reported differences in the intake of energy and nutrients among seasons

	Males and Females	Males				Females								
		
	Akimoto^a^	Sasaki	Owaki	Nozue	Ishiwaki	Sasaki	Tokudome	Owaki	Amano^a^	Nozue	Ishiwaki	Miyai^b^	Miyai^c^	Minari
	(32)	(2)	(4)	(24)	(30)	(2)	(3)	(4)	(29)	(24)	(30)	(25)	(25)	(31)
Energy	n.s.	n.s.	n.s.	n.s.	n.s.	n.s.	^**^	n.s.	n.s.	n.s.	n.s.	n.s.	n.s.	^*^
Protein	n.s.	n.s.	n.s.	n.s.	^**^	n.s.	^**^	n.s.	n.s.	n.s.	n.s.	—	—	^*^
Animal protein	—	—	n.s.	n.s.	—	—	—	n.s.	—	n.s.	—	—	—	—
Fat	n.s.	n.s.	^*^	^**^	n.s.	n.s.	^***^	n.s.	n.s.	n.s.	n.s.	—	—	^*^
Animal fat	—	—	n.s.	n.s.	—	—	—	n.s.	—	n.s.	—	—	—	—
SFA	n.s.	—	n.s.	—	—	—	^**^	n.s.	n.s.	—	—	—	—	—
MUFA	n.s.	—	^**^	—	—	—	^*^	^*^	n.s.	—	—	—	—	—
PUFA	n.s.	—	^**^	—	—	—	^**^	^**^	^*^	—	—	—	—	—
n-6 PUFA	—	—	—	—	—	—	^**^	—	—	—	—	—	—	—
n-3 PUFA	—	—	—	—	—	—	^***^	—	—	—	—	—	—	—
Cholesterol	n.s.	n.s.	n.s.	^*^	—	n.s.	n.s.	n.s.	n.s.	n.s.	—	—	—	—
Carbohydrate	n.s.	n.s.	n.s.	n.s.	—	^*^	n.s.	n.s.	^*^	n.s.	—	—	—	^*^
Total fiber	n.s.	—	^**^	n.s.	—	—	^***^	^*^	n.s.	^***^	—	—	—	^*^
Soluble dietary fiber	—	—	n.s.	n.s.	—	—	^***^	^*^	n.s.	^***^	—	—	—	—
Insoluble dietary fiber	—	—	^**^	^*^	—	—	^***^	^*^	n.s.	^***^	—	—	—	—
Vitamin A	—	—	n.s.	n.s.	—	—	^**^	n.s.	n.s.	^**^	—	—	—	^*^
Vitamin D	—	—	n.s.	n.s.	—	—	n.s.	n.s.	n.s.	^**^	—	—	—	—
Vitamin E	—	—	^**^	^*^	—	—	^***^	^*^	n.s.	n.s.	—	—	—	—
Vitamin K	—	—	—	^**^	—	—	—	—	^**^	^*^	—	—	—	—
Thiamine	—	n.s.	n.s.	n.s.	—	n.s.	—	n.s.	n.s.	n.s.	—	—	—	^*^
Riboflavin	—	n.s.	n.s.	n.s.	—	n.s.	—	n.s.	^*^	n.s.	—	—	—	^*^
Niacin	—	n.s.	—	n.s.	—	n.s.	—	—	n.s.	n.s.	—	—	—	—
Vitamin B_6_	—	—	—	n.s.	—	—	—	—	n.s.	n.s.	—	—	—	—
Vitamin B_12_	—	—	—	n.s.	—	—	—	—	n.s.	n.s.	—	—	—	—
Folate	—	—	—	n.s.	n.s.	—	—	—	^**^	^**^	n.s.	—	—	—
Pantothenic acid	—	—	—	n.s.	—	—	—	—	^**^	n.s.	—	—	—	—
Vitamin C	—	^***^	^**^	^*^	^***^	^***^	^***^	^**^	^**^	n.s.	^***^	—	—	^*^
Sodium/Salt	—	n.s.	^**^	n.s.	^**^	^**^	—	^*^	n.s.	^**^	^*^	—	—	^*^
Potassium	—	n.s.	^**^	^*^	—	n.s.	^***^	^**^	^*^	^*^	—	—	—	—
Calcium	—	n.s.	n.s.	n.s.	n.s.	^*^	^***^	n.s.	^**^	n.s.	n.s.	—	—	^*^
Magnesium	—	—	^*^	^*^	—	—	^***^	^*^	n.s.	n.s.	—	—	—	—
Phosphorus	—	n.s.	n.s.	n.s.	—	n.s.	n.s.	n.s.	^**^	n.s.	—	—	—	—
Iron	—	^**^	^*^	n.s.	n.s.	^***^	^***^	n.s.	n.s.	n.s.	n.s.	—	—	^*^
Zinc	—	—	n.s.	n.s.	—	—	^***^	n.s.	n.s.	n.s.	—	—	—	—
Copper	—	—	n.s.	n.s.	—	—	^***^	n.s.	n.s.	n.s.	—	—	—	—

SFA, Saturated fatty acids. MUFA, Monounsaturated fatty acids. PUFA, Polyunsaturated fatty acids.

^*^P < 0.05, ^**^P < 0.01, ^***^P < 0.001.

n.s. represents the lack of a significant difference in intake among the seasons.

“—” represents the nutrient was not surveyed in the study.

^a^Energy-adjusted value.

^b^Females with normal weight.

^c^Females with overweight or obesity.

**Table 4.** Reported differences in the intake of food groups among the four seasons

	Males and Females	Males		Females			
		
	Tatsumi	Sasaki	Nozue	Sasaki	Miyai^a,b^	Miyai^a,c^	Nozue
	(26)	(2)	(24)	(2)	(25)	(25)	(24)
Grains	—	n.s.	n.s.	n.s.	n.s.	n.s.	n.s.
Potatoes	^***^	^***^	n.s.	^***^	—	—	^**^
Sugar and sweeteners	—	n.s.	n.s.	n.s.	—	—	n.s.
Beans	^***^	n.s.	^*^	^*^	—	—	n.s.
Nuts and seeds	—	n.s.	n.s.	n.s.	—	—	n.s.
Vegetables	^***^	^***^	^***^	^***^	—	—	^***^
Fruits	^***^	^***^	^*^	^***^	—	—	^***^
Mushrooms	—	^***^	^**^	^***^	—	—	^**^
Seaweeds	—	^*^	^*^	n.s.	—	—	^**^
Fish and shellfish	—	n.s.	^*^	n.s.	—	—	^*^
Meats	—	n.s.	n.s.	n.s.	—	—	n.s.
Eggs	—	n.s.	n.s.	n.s.	—	—	n.s.
Dairy	—	n.s.	n.s.	^***^	—	—	^*^
Fats and oils	—	^**^	^**^	^**^	n.s.	n.s.	n.s.
Seasonings and spices	—	n.s.	n.s.	n.s.	—	—	n.s.

^*^P < 0.05, ^**^P < 0.01, ^***^P < 0.001.

“—” represents the food groups not surveyed in the study.

n.s. represents the lack of a significant difference in intake among the seasons.

^a^Energy-adjusted value.

^b^Females with normal weight.

^c^Females with overweight or obesity.

### Differences in mean intakes between two seasons

Meta-analyses were performed to investigate differences in mean intakes between two seasons (ie, spring vs summer, spring vs fall, spring vs winter, summer vs fall, summer vs winter, and fall vs winter) on energy, 32 nutrients, and 15 food groups investigated in minimum two studies. Table 5 shows the pooled mean differences, *P*-values, 95% CIs, and *I*^2^ index between two seasons for nutrients, and Table 6 shows them for food groups. Most nutrients and food groups had no significant mean differences in all comparisons or had significant mean differences in only one comparison. Significant mean differences were observed for potatoes, vegetables, and fruits in five out of the six comparisons (their forest plots are shown in eFigure 1, eFigure 2, and eFigure 3). Potato intake was higher in fall and winter, vegetable intake was higher in summer, and fruit intake was higher in fall. The biggest differences were as follows: 20.1 g/day more potato intake in fall than spring, 101 g/day more vegetable intake in summer than spring, and 60 g/day more fruit intake in fall than spring. Although significant seasonal differences in vitamin C were reported in most studies, the pooled mean differences were lower in summer than in fall and winter by 18 mg/day and 13 mg/day, respectively, and the other seasonal comparisons did not show significant differences. Most comparisons showed high heterogeneity, with *I*^2^ of more than 50%. The results of sensitivity analyses were consistent for most nutrients and food groups, but the mean differences became not significant for soluble dietary fiber between summer and fall, vitamin E between fall and winter, and zinc between spring and winter (data not shown).

**Table 5.** Differences in mean intakes of energy and nutrients between two seasons

		Spring vs Summer		Spring vs Fall		Spring vs Winter		Summer vs Fall		Summer vs Winter		Fall vs Winter	

		Mean difference [95% CI]	*I* ^2^	Mean difference [95% CI]	*I* ^2^	Mean difference [95% CI]	*I* ^2^	Mean difference [95% CI]	*I* ^2^	Mean difference [95% CI]	*I* ^2^	Mean difference [95% CI]	*I* ^2^
Energy	kcal/day	30 [−29 to 88]	100	34 [−13 to 80]	99	20 [−7 to 46]	98	0 [−63 to 62]	100	−10 [−66 to 46]	100	−18 [−67 to 32]	99
Protein	g/day	2.9 [1.2–4.7]	85	1.7 [−0.2 to 3.5]	85	1.0 [−0.9 to 2.9]	85	−1.1 [−3.4 to 1.2]	90	−1.9 [−4.0 to 0.1]	88	−1.1 [−3.8 to 1.5]	92
Animal Protein	g/day	1.3 [−0.1 to 2.8]	16			0.8 [−1.5 to 3.0]	65			−0.6 [−2.0 to 0.8]	0		
Fat	g/day	1.9 [−0.5 to 4.4]	93	3.1 [0.0–6.2]	95	1.4 [−0.1 to 3.0]	80	0.7 [−2.5 to 3.9]	96	−0.4 [−3.2 to 2.4]	94	−2.0 [−5.6 to 1.6]	96
Animal Fat	g/day	1.8 [0.6–3.0]	0			1.0 [−0.8 to 2.7]	51			−0.9 [−3.3 to 1.6]	77		
SFA	g/day	0.6 [−0.8 to 2.1]	81	−0.1 [−0.7 to 0.5]	0	0.1 [−0.8 to 1.0]	47	−0.7 [−2.4 to 0.9]	85	−0.6 [−1.5 to 0.2]	45	0.2 [−0.8 to 1.2]	57
MUFA	g/day	0.5 [−1.8 to 2.7]	90	0.6 [−0.2 to 1.4]	26	0.8 [0.0–1.6]	25	0.2 [−2.4 to 2.7]	92	0.2 [−1.8 to 2.2]	88	0.2 [−0.5 to 0.8]	0
PUFA	g/day	0.4 [−1.0 to 1.8]	78	0.8 [−0.2 to 1.9]	60	1.2 [0.3–2.0]	40	0.4 [−1.6 to 2.4]	89	0.6 [−0.9 to 2.2]	82	0.3 [−0.6 to 1.2]	49
Cholesterol	mg/day	24 [2–46]	99	13 [−15 to 41]	99	28 [11–46]	99	−3 [−25 to 18]	99	4 [−8 to 17]	97	15 [3–28]	96
Carbohydrate	g/day	5 [−2 to 13]	97	0 [−7 to 7]	96	2 [−3 to 6]	91	−7 [−15 to 1]	97	−4 [−10 to 3]	95	3 [−5 to 11]	96
Total fiber	g/day	1.4 [−0.3 to 3.1]	91	0.4 [−0.7 to 1.5]	74	−0.3 [−1.5 to 0.9]	79	−1.9 [−3.4 to −0.3]	88	−1.7 [−3.4 to 0.1]	91	0 [−1.1 to 1.2]	71
Soluble dietary fiber	g/day	0.1 [−0.3 to 0.5]	43	0.0 [−0.3 to 0.3]	0	−0.1 [−0.5 to 0.3]	43	−0.3 [−0.6 to 0.0]	0	−0.2 [−0.5 to 0.1]	0	0.1 [−0.2 to 0.4]	0
Insoluble dietary fiber	g/day	0.8 [0.1–1.5]	51	0.4 [−0.7 to 1.5]	72	0.0 [−1.1 to 1.1]	79	−0.8 [−1.7 to 0.0]	61	−0.7 [−1.2 to −0.2]	18	0.4 [−0.2 to 0.9]	4
Vitamin A	µgRE/day	27 [−41 to 95]	99	−64 [−136 to 8]	99	−56 [−187 to 75]	100	−124 [−200 to −49]	99	−82 [−176 to 12]	100	95 [−39 to 229]	100
Vitamin D	µg/day	−0.7 [−2.0 to 0.6]	75	0.1 [−0.7 to 0.8]	0	0.4 [−0.6 to 1.4]	62	−0.2 [−1.0 to 0.6]	21	1.0 [0.4–1.6]	0	1.0 [0.2–1.7]	21
Vitamin E	mg/day	0.1 [−0.9 to 1.0]	68	0.2 [−0.3 to 0.7]	0	0.8 [0.2–1.4]	30	0.3 [−0.9 to 1.5]	82	0.8 [0.0–1.6]	68	0.6 [0.1–1.1]	0
Vitamin K	µg/day	46 [37–56]	74			−12 [−51 to 28]	98			−58 [−90 to −26]	98		
Thiamine	mg/day	0.06 [−0.06 to 0.17]	0	0.05 [−0.08 to 0.17]	0	0.00 [−0.13 to 0.14]	0	−0.01 [−0.13 to 0.11]	0	−0.05 [−0.17 to 0.08]	0	−0.05 [−0.18 to 0.09]	0
Riboflavin	mg/day	0.04 [−0.10 to 0.18]	0	0.06 [−0.09 to 0.21]	0	0.02 [−0.13 to 0.17]	0	0.01 [−0.15 to 0.17]	20	−0.04 [−0.18 to 0.10]	0	0.00 [−0.15 to 0.16]	0
Niacin	mg/day	0.4 [−0.6 to 1.5]	64	0.6 [−0.1 to 1.3]	0	0.3 [−0.3 to 0.9]	0	−0.2 [−2.0 to 1.5]	83	−0.2 [−1.1 to 0.7]	54	−0.1 [−0.8 to 0.6]	0
Vitamin B_6_	mg/day	0.14 [−0.08 to 0.35]	0			0.06 [−0.16 to 0.27]	0			−0.09 [−0.29 to 0.12]	0		
Vitamin B_12_	mg/day	1.3 [0.5–2.1]	0			0.8 [0.0–1.6]	0			−0.5 [−1.3 to 0.3]	0		
Folate	µg/day	19 [−4 to 42]	98	2 [−24 to 27]	98	−5 [−39 to 28]	99	−25 [−83 to 34]	100	−24 [−50 to 2]	99	19 [−22 to 60]	99
Pantothenic acid	mg/day	0.24 [−0.63 to 1.10]	71			0.24 [−0.29 to 0.77]	25			−0.21 [−0.62 to 0.19]	0		
Vitamin C	mg/day	6 [−2 to 15]	98	−12 [−29 to 5]	99	−6 [−22 to 9]	99	−18 [−29 to −8]	99	−13 [−24 to −1]	99	6 [−9 to 21]	99
Salt equivalent	g/day	−0.1 [−0.7 to 0.6]	69	0.4 [−0.3 to 1.1]	69	0.0 [−0.5 to 0.6]	57	0.4 [−0.2 to 0.9]	55	0.1 [−0.6 to 0.7]	70	−0.2 [−0.8 to 0.4]	66
Potassium	mg/day	−58 [−178 to 62]	100	3 [−163 to 168]	100	6 [−146 to 158]	100	21 [−231 to 274]	100	64 [−88 to 216]	100	61 [−46 to 168]	100
Calcium	mg/day	31 [1–61]	99	−3 [−28 to 21]	99	24 [−38 to 86]	100	−17 [−42 to 8]	99	−8 [−71 to 55]	100	27 [−48 to 103]	100
Magnesium	mg/day	1 [−12 to 13]	97	−1 [−15 to 13]	97	13 [3–23]	96	−6 [−32 to 19]	99	12 [−4 to 28]	98	14 [5–24]	93
Phosphorous	mg/day	27 [−9 to 62]	99	14 [−22 to 49]	99	42 [12–73]	99	−6 [−70 to 58]	100	16 [−13 to 45]	99	25 [−13 to 63]	99
Iron	mg/day	0.5 [0.2–0.8]	0	0.2 [−0.1 to 0.5]	0	−0.1 [−0.5 to 0.4]	47	−0.2 [−0.7 to 0.2]	47	−0.5 [−1.0 to −0.1]	49	−0.3 [−0.7 to 0.2]	42
Zinc	mg/day	0.4 [−0.1 to 0.9]	44	0.1 [−0.4 to 0.5]	0	0.4 [0.0–0.8]	0	−0.4 [−1.1 to 0.3]	55	−0.1 [−0.7 to 0.4]	51	0.3 [−0.1 to 0.8]	0
Copper	mg/day	0.0 [−0.1 to 0.2]	0	0.0 [−0.2 to 0.1]	0	0.0 [−0.2 to 0.2]	0	−0.1 [−0.3 to 0.1]	0	0.0 [−0.2 to 0.1]	0	0.1 [−0.2 to 0.3]	0

CI, confidence interval; MUFA, monounsaturated fatty acids; PUFA, polyunsaturated fatty acids; RE, retinol equivalents; SFA, saturated fatty acids. Mean differences were calculated by subtracting the intake in the right season from the intake in the left season. Meta-analysis was conducted when a minimum of two studies reported the same outcomes. Vitamin C intakes in Nozue et al. were excluded from meta-analyses because their standard deviations were abnormally large. (See eTable 2)

**Table 6.** Differences in mean intakes of food groups between two seasons

		Spring vs Summer		Spring vs Fall		Spring vs Winter		Summer vs Fall		Summer vs Winter		Fall vs Winter	

		Mean difference [95% CI]	*I* ^2^	Mean difference [95% CI]	*I* ^2^	Mean difference [95% CI]	*I* ^2^	Mean difference [95% CI]	*I* ^2^	Mean difference [95% CI]	*I* ^2^	Mean difference [95% CI]	*I* ^2^
Grains	g/day	−5 [−28 to 19]	98	23 [−7 to 53]	98	13 [2–23]	92	34 [−19 to 86]	100	17 [−8 to 43]	99	−16 [−51 to 19]	99
Potatoes	g/day	6.2 [−3.7 to 16.1]	98	−20.1 [−30.6 to −9.5]	97	−13.3 [−18.3 to −8.4]	90	−19.4 [−26.4 to −12.3]	94	−19.5 [−29.3 to −9.7]	98	4.8 [0.8–8.9]	81
Sugar	g/day	0.3 [−0.6 to 1.2]	0			0.3 [−1.4 to 2.1]	76			0.1 [−1.9 to 2.0]	77		
Beans	g/day	4.2 [0.1–8.2]	81	1.6 [−0.1 to 3.3]	0	1.6 [−5.7 to 8.8]	94	−4.1 [−8.6 to 0.3]	83	−2.4 [−11.9 to 7.1]	97	−6.1 [−9.8 to −2.3]	74
Nuts and seeds	g/day	0.0 [−0.6 to 0.7]	0			−0.1 [−1.1 to 0.8]	49			−0.3 [−1.2 to 0.7]	34		
Vegetables	g/day	−101 [−152 to −49]	100	15 [10–19]	58	−33 [−71 to 6]	100	78 [32–125]	99	68 [35–101]	99	−17 [−33 to −1]	96
Fruits	g/day	−53 [−71 to −35]	97	−60 [−68 to −53]	82	−33 [−60 to −6]	99	−12 [−23 to −2]	91	20 [−2 to 42]	98	49 [47–52]	0
Mushrooms	g/day	3.4 [−2.8 to 9.7]	97			−1.7 [−5.7 to 2.3]	92			−4.9 [−7.4 to −2.4]	80		
Seaweeds	g/day	−4.3 [−7.4 to −1.2]	79			3.4 [1.2–5.5]	72			7.9 [3.8–12.0]	88		
Fish and shellfish	g/day	11.6 [−3.4 to 26.7]	97			3.7 [−10.9 to 18.2]	97			−7.9 [−13.5 to −2.3]	81		
Meats	g/day	−5.1 [−12.1 to 1.8]	92			−4.8 [−11.7 to 2.2]	91			0.4 [−1.6 to 2.4]	0		
Eggs	g/day	1.6 [−1.3 to 4.6]	70			2.4 [0.8–4.0]	13			1.0 [−1.3 to 3.4]	56		
Dairy	g/day	−16 [−20 to −12]	12			11 [−2 to 24]	92			28 [19–37]	83		
Fats and oils	g/day	−1.9 [−2.8 to −1.0]	35	−1.0 [−3.4 to 1.5]	87	0.5 [−0.6 to 1.6]	59	1.7 [0.9–2.6]	0	2.4 [1.6–3.2]	21	0.6 [−0.4 to 1.6]	30
Seasonings and spices	g/day	0.3 [−3.0 to 3.6]	75			−4.4 [−10.5 to 1.7]	93			−4.7 [−10.7 to 1.2]	93		

CI, confidence interval.

Mean differences were calculated by subtracting the intake in the right season from the intake in the left season. Meta-analysis was conducted when a minimum of two studies reported the same outcomes.

## DISCUSSION

This study summarized seasonal variation in dietary intake from the available studies conducted in Japan. The reported seasonal variations were inconsistent across the included study for most food groups and nutrients. In meta-analyses, food groups tended to show significant mean differences more than nutrients, and relatively distinct seasonal variations were observed for vegetables (101 g/day difference) and fruits (60 g/day difference), although the observed differences in the pooled mean intakes were attributed to high heterogeneity of the included studies, not just seasonal variations. Our findings would be helpful to interpret results obtained from dietary survey, for example, the Japan National Health and Nutrition Survey, which has been conducted only in fall for more than the past 50 years.^33^ Moreover, considering the current available dietary recommendations for Japanese on vegetable (350 g/day) and fruits (200 g/day),^34^ observed biggest mean differences between seasons were noteworthy; thus, it would be recommended to take into account seasonal differences in dietary intakes when designing studies if these sizes of differences were relevant. In contrast, several studies have suggested that within-individual variations other than seasonality were greater.^3^^,^^35^^–^^37^ Thus, to assess habitual dietary intake accurately, considering potential factors which can be sources of variations in dietary intake, including seasons, would be recommended.

Although it is difficult to interpret our results with respect to those of previous studies conducted in other countries because of the different variations in the living conditions in countries, seasonal variations were observed in the intake of vegetables and fruits. A meta-analysis of six studies from five countries (Japan, China, Spain, Germany, and Finland) showed that vegetable intake increased from spring to summer, and decreased from summer to fall, while fruit intake increased from summer to fall.^13^ Another study conducted in the United States showed that intakes of fruits and vegetables assessed using biomarkers were higher in summer, though there was no difference in intakes of them assessed using a food frequency questionnaire.^7^ Seasonal variations in the consumption of fruits and vegetables are considered to be relatively substantial because they are mainly grown outside and are affected by the seasonality of temperature and precipitation. In addition, compared with other food groups, fruits and vegetables are considered seasonal foods.^38^ Thus, yearly agricultural cycles and consumer trends may influence seasonality in fruit and vegetable intake in a similar manner across countries.

The characteristics of the included studies made it challenging to conclude the pooled mean differences were attributed to just distinct seasons, although their reporting quality was similar. Only Owaki et al^4^ used the 24-hour recall method, and they reported that vitamin C intake in spring was the highest, whereas that in fall was the highest in most studies. Tokudome et al^3^ and Minari et al^31^ reported significant differences for more than 80% of the nutrients examined, which might have been attributed to the female exclusive study participants, not general people. The other two studies which invited only female participants had unique aims to evaluate the accuracy of dietary records supplemented with photography^29^ and to investigate the seasonal variation of snack intake among female with obesity.^25^ Tatsumi et al,^26^ Akimoto, et al,^32^ and Ishiwaki et al^30^ reported a limited number of nutrients and food groups most relevant to their study aims. Sasaki et al^2^ reported relatively many nutrients and food groups because of the nature of the validation study for a food frequency questionnaire. Although Nozue et al^24^ also reported similar number of nutrients and food groups as Sasaki et al^2^ for three seasons, they reported abnormally large means and standard deviations of vitamin C, which were excluded from our meta-analysis. The observed results should be interpreted with caution and might not reflect the true seasonal variation in dietary intake in Japan because each nutrient and food group have been examined in different datasets obtained from the participants with specific characteristics.

The included studies may only partially represent the Japanese population. The included studies covered the northernmost to the southernmost part of Japan, but the number of prefectures covered approximately half of the 47 prefectures, which might have resulted in reduced regional variation in dietary intake. Eight^2^^–^^4^^,^^24^^–^^26^^,^^29^^,^^30^ of 10 studies designated seasons by months, and their definitions were similar. There may be differences in temperature and precipitation even during the same month for different survey areas.^1^ The definition of seasons by months and the description of seasons, such as temperature and precipitation, may lead to better comparability within Japan, as well as with studies conducted outside of Japan. In addition, four^3^^,^^25^^,^^29^^,^^31^ of the included studies enrolled only females, even though previous studies concluded that seasonal variations in dietary intake depended on sex.^9^^,^^39^ Furthermore, even inside Japan or in the same prefecture, dietary intake might depend on individuals’ demographic characteristics related to local food production. For example, a previous study conducted in a single prefecture in Japan showed that people living in rural areas had a higher frequency of vegetables than people living in urban areas due to the differences in vegetable cultivation.^40^

The time periods of the studies included in this study varied from 1992 to 2014. Possible changes over time in nutrient content in foods and dietary pattern might have affected the results. For example, possible decreases in nutritional content due to modern agricultural methods for higher yields could mask the seasonal variations.^41^ In addition, we might have failed to capture recent seasonal variations (eg, from 2015 to 2024). Several studies have shown that the dietary intake pattern of the Japanese population has changed over time,^42^^,^^43^ and the total import value of agricultural products gradually increased.^44^ These changes might have affected individual’s food choices, but theoretically, they would bring less seasonal variation in dietary intake at the population level because various food could be purchased irrelevant to seasons.

This study has several limitations. First, grey literature and additional databases other than PubMed and Ichushi-Web were not used to search for eligible studies because of practical constraints. However, we developed comprehensive search terms aimed to include as many potential studies as possible in English and Japanese. Second, our inclusion criteria may have resulted in fewer eligible studies. We limited our review to the studies that used dietary records or 24-hour recalls to collect absolute dietary intake data and excluded studies that used other dietary methods, such as a food frequency questionnaire, because they predefined food items on the list which may mask the seasonal variation in dietary intakes. Third, almost all the included studies utilized the standard tables of food composition in Japan,^16^ which have different nutrient values in different seasons only for spinach and bonito. For example, raw spinach has different vitamin C values (20 mg/100 g in summer and 60 mg/100 g in winter). Seasonal variations in the nutrient content were not considered for most foods in the tables. This may have reduced the apparent seasonal differences in nutrient intake. Fourth, we assumed dietary intakes correlated each season with 0.6 correlation coefficients based on previous study conducted in the United States,^27^ as we did not have crude data from each study or available reports on seasonal correlation of dietary intake in Japan. As the results from sensitivity analyses show, the significance in mean differences would not be likely to change considerably, but careful interpretation is needed. Finally, we did not conduct a subgroup analysis by sex and age group owing to the small number of eligible studies, particularly those investigating food groups.

In conclusion, the results of this systematic review and meta-analyses suggest that seasons might influence the intakes of some nutrients and food groups, although the results must be interpreted with caution because the number of studies were small and the included studies had limited representativeness of the Japanese population. In addition, the observed differences in the pooled mean intakes between seasons were highly attributed to the heterogeneity of the included studies, not just seasonal variations. However, our review contributes to summarizing the existing study results on seasonal variations in dietary intake in Japan, which have been said to exist but have not thoroughly been studied. We revealed a need for further studies with nationally representative participants to understand seasonal variations in dietary intake in Japan.

## SUPPLEMENTARY MATERIAL

eTable 1. Risk of bias assessment^a^

eTable 2. Mean intake of energy and nutrients in each season

eTable 3. Mean intake of food groups in each season

eFigure 1. Forest plot of the pooled mean differences in potato intakes. Spring vs Summer (A), Spring vs Fall (B), Spring vs Winter (C), Summer vs Fall (D), Summer vs Winter (E), Fall vs Winter (F). Mean differences were calculated by subtracting the intake in the right season from the intake in the left season. M indicates males and F indicates females.

eFigure 2. Forest plot of the pooled mean differences in vegetable intakes. Spring vs Summer (A), Spring vs Fall (B), Spring vs Winter (C), Summer vs Fall (D), Summer vs Winter (E), Fall vs Winter (F). Mean differences were calculated by subtracting the intake in the right season from the intake in the left season. M indicates males and F indicates females.

eFigure 3. Forest plot of the pooled mean differences in fruit intakes. Spring vs Summer (A), Spring vs Fall (B), Spring vs Winter (C), Summer vs Fall (D), Summer vs Winter (E), Fall vs Winter (F). Mean differences were calculated by subtracting the intake in the right season from the intake in the left season. M indicates males and F indicates females.
