# Supplementary material for: Seasonal Variation in the Intake of Food Groups and Nutrients in Japan: A Systematic Review and Meta-analysis
Source: J Epidemiol. 2025 Feb 5;35(2):53–62. doi: 10.2188/jea.JE20240139 (PMC11706675; doi:10.2188/jea.JE20240139)
Supplement: Supplementary file 1 [file je-35-053-s001.pdf]

eTable 1. Risk of bias assessment[a]

No.	Item	References									
		2	3	4	24	25	26	29	30	31	32
1	Were the aims/objectives of the study clear?	1	1	1	1	1	1	1	1	1	1
2	Was dietary intake examined for more than 90% of the same individuals over four seasons?	1	1	1	1	1	1	1	1	1	1
3	Was the sample size justified?	1[b]	0	0	0	0	0	0	0	0	0
4	Was the target/reference population clearly defined at least for sex, age, research area, and recruitment method? (Is it clear who the research was about?)	1[b]	1	1	1	1	1	1	0	0	1
5	Was the sample frame appropriate for investigating the seasonal variation among the Japanese population (i.e. both sexes and not too specific)?	1[b]	0	1	0	0	1	0	1	0	1
6	Was the selection process likely to select subjects/participants that were representative of the target/reference population under investigation?	1[b]	1	1	0	0	1	1	1	0	1
7	Were measures undertaken to address and categorise non-responders?	0	0	0	0	0	0	0	0	0	0
8	Was the definition of seasons defined?	1[b]	1	1	1	1	1	1	1	0	0
9	Was the dietary intake weighed and checked by the trained research staff?	1	1	1	1	0	1	0	1	1	1
10	Is it clear what was used to determined statistical significance and/or precision estimates on dietary intake in each season? (e.g. p values, CIs)	1	1	1	1	1	1	1	1	1	1
11	Were the dietary survey methods (the number of dietary survey days and whether they were consecutive) and statistical methods sufficiently described to enable them to be repeated?	1	1	1	1	0	1	1	1	1	1
12	Were the basic data (at least age and sex) adequately described?	1[b]	1	1	1	1	1	1	1	1	1
13	Does the response rate raise concerns about non-response bias?	N/A	N/A	1	1	1	N/A	0	N/A	N/A	N/A
14	If appropriate, was information about non-responders described?	0	0	0	0	1	0	1	0	0	0
15	Were the results internally consistent?	1	1	1	1	1	1	1	1	1	1
16	Were the results of the analyses described in the methods presented?	1	1	1	1	1	1	1	1	1	1
17	Was the number of survey days multiplied by the number of participants over 150?	1	1	0	1	0	1	1	1	1	1
	Total (Maximum 17 points)	14	12	13	12	10	13	12	12	9	12

[a]Quality assessment was conducted by using AXIS tool with modifications. N/A was converted to zero in the total score calculation.

[b] This information was extracted from the cohort profile paper published in the same issue. (Tsugane S, Sasaki S, Kobayashi M, et al. Validity and reproducibility of the self-administered food frequency questionnaire in the JPHC Study Cohort I: study design, conduct and participant profiles. *J Epidemiol*. 2003 Jan;13:S2–S12.)

eTable 2. Mean intake of energy and nutrients in each season

			Season											
			Spring		Summer		Fall		Winter		ALL			
	Unit	Population	mean	SD	mean	SD	mean	SD	mean	SD	mean	SD	P-value	P-value detail
Energy														
Sasaki (2)	kcal/day	Males	2447	457	2466	497	2491	449	2415	413	2455	-	0.343	Two-way ANOVA adjusted for area difference
Sasaki (2)	kcal/day	Females	1892	365	1883	352	1900	311	1861	311	1884	-	0.657	Two-way ANOVA adjusted for area difference
Tokudome (3)	kcal/day	Females	1811	348	1792	362	1852	346	1825	352	1820	-	<0.01	ANOVA
Owaki (4)	kcal/day	Males	2232	558	2206	547	2087	477	2135	468	2165	345	n.s.	ANOVA
Owaki (4)	kcal/day	Females	1808	359	1781	405	1714	326	1769	375	1768	323	n.s.	ANOVA
Nozue (24)	kcal/day	Males	2413	525	2433	545	N/A	N/A	2333	464	2393	-	-	Paired t tests were conducted for each two seasons
Nozue (24)	kcal/day	Females	1923	420	1894	339	N/A	N/A	1917	410	1911	-	-	Paired t tests were conducted for each two seasons
Miyai (25)	kcal/day	Normal weight Females	1667	297	1575	206	1652	265	1689	268	1646	155	0.363	Paired friedman test
Miyai (25)	kcal/day	Overweight Females	1656	262	1893	207	1684	284	1707	296	1735	167	0.284	Paired friedman test
Amano (29)	kcal/day	Females	1647	314	1401	303	1639	393	1536	250	1556	-	n.s.	ANOVA
Ishiwaki (30)	kcal/day	Males	2396	456	2407	488	2346	471	2366	493	2379	404	>0.05	ANOVA
Ishiwaki (30)	kcal/day	Females	1880	344	1864	391	1867	358	1871	363	1870	307	>0.05	ANOVA
Minari (31)	kcal/day	Females	1827	69	1615	102	1605	73	1852	279	1725	-	<0.05	Steel-Dwass's multiple comparison test
Akimoto (32)	kcal/day	Males and Females	1911	549	1887	454	1935	478	1959	406	1923	-	0.48	Mixed effect model
Protein														
Sasaki (2)	g/day	Males	95.3	16.5	95.5	17.5	97.2	16.8	97.2	18.6	96.3	-	0.495	Two-way ANOVA adjusted for area difference
Sasaki (2)	g/day	Females	78.8	14.3	77.4	13.7	78.7	12.9	79.1	15.4	78.5	-	0.634	Two-way ANOVA adjusted for area difference
Tokudome (3)	g/day	Females	73.6	17.9	73.1	18.1	75.7	15.9	74.8	17.3	74.3	-	<0.01	ANOVA
Owaki (4)	g/day	Males	83.2	22.5	83.7	26.5	81	23.7	80.9	19.2	82.2	15.1	n.s.	ANOVA
Owaki (4)	g/day	Females	73.0	18.2	71.7	18.2	68.9	17.7	71.2	20.8	71.2	16.1	n.s.	ANOVA
Nozue (24)	g/day	Males	90.3	20.3	87.3	21.8	N/A	N/A	85.8	16.9	87.8	-	-	Paired t tests were conducted for each two seasons
Nozue (24)	g/day	Females	76.4	18.0	71.7	17.1	N/A	N/A	73.4	16.8	73.8	-	-	Paired t tests were conducted for each two seasons
Amano (29)	g/1000 kcal	Females	37.9	5.8	37.2	5.0	37.6	5.5	37	5.1	58.1	-	n.s.	ANOVA
Ishiwaki (30)	g/day	Males	92.4	19.7	88.9	20.5	87.1	18.9	89.0	20.3	89.3	16.3	<0.01	ANOVA
Ishiwaki (30)	g/day	Females	76.5	15.8	73.8	18.1	73.9	16.7	74.3	17	74.6	14.1	>0.05	ANOVA
Minari (31)	g/day	Females	68.9	4.7	63.1	4.8	62.9	3.7	75.3	16.4	67.6	-	<0.05	Steel-Dwass's multiple comparison test
Akimoto (32)	g/1000 kcal	Males and Females	36.0	5.0	35.6	4.6	36.0	5.0	36.0	5.0	35.9	-	1.00	Mixed effect model
Animal protein														
Owaki (4)	g/day	Males	38.6	17.4	39.1	22.5	40.9	19.7	40.3	16.5	39.7	13.7	n.s.	ANOVA
Owaki (4)	g/day	Females	34.1	14.3	33.5	14.9	33.4	15.6	34.7	17.7	33.9	12.8	n.s.	ANOVA
Nozue (24)	g/day	Males	49.1	15.8	46.8	17.5	N/A	N/A	46.1	13.9	47.3	-	-	Paired t test
Nozue (24)	g/day	Females	39.5	13.9	36.5	15.1	N/A	N/A	37.0	13.5	37.7	-	-	Paired t test

eTable 2. *Cont.*

	Unit	Population	Season: Spring mean	SD	Summer mean	SD	Fall mean	SD	Winter mean	SD	ALL mean	SD	P-value	P-value detail
Fat														
Sasaki (2)	g/day	Males	57	11.8	58.1	11.7	58.3	13.7	58.3	14.1	57.9	-	0.814	Two-way ANOVA adjusted for area difference
Sasaki (2)	g/day	Females	52	11.3	52.9	11	50.4	9.2	52.5	11.4	52.0	-	0.187	Two-way ANOVA adjusted for area difference
Tokudome (3)	g/day	Females	55.9	18.6	54.9	19.4	58.7	19.1	55.5	18.5	56.3	-	<0.001	ANOVA
Owaki (4)	g/day	Males	55.1	23.5	54.8	21.7	47.4	15.9	49.5	19.4	54.5	14.9	<0.05	ANOVA
Owaki (4)	g/day	Females	50.3	19.4	50.0	20.4	44.6	15.2	47.7	19.9	48.2	16.0	n.s.	ANOVA
Nozue (24)	g/day	Males	60.5	21.2	63.4	24.7	N/A	N/A	55.6	21.0	59.8	-	-	Paired t tests were conducted for each two seasons
Nozue (24)	g/day	Females	51.3	19.4	50.1	15.3	N/A	N/A	48.9	18.2	50.1	-	-	Paired t tests were conducted for each two seasons
Amano (29)	g/1000 kcal	Females	35.1	6.0	32.9	5.4	33.9	5.8	34.2	5.7	34.3	-	n.s.	ANOVA
Ishiwaki (30)	g/day	Males	58.9	16.7	59.5	18.9	57.4	20.4	58.4	19.0	58.5	14.5	>0.05	ANOVA
Ishiwaki (30)	g/day	Females	52.2	15.8	50.7	14.9	50.3	17.6	50.8	15.5	51.0	12.3	>0.05	ANOVA
Minari (31)	%E	Females	27.7	1.0	26.6	1.6	23.5	2.1	28.8	4.2	26.7	-	<0.05	Steel-Dwass's multiple comparison test
Akimoto (32)	g/1000 kcal	Males and Females	32.7	6.3	31.4	5.9	32.2	5.9	31.8	6.3	32.0	-	0.35	Mixed effect model
Animal fat														
Owaki (4)	g/day	Males	19.8	15.6	16.7	11.8	17.0	10.8	20.2	12.5	18.4	9.8	n.s.	ANOVA
Owaki (4)	g/day	Females	18.5	12.7	16.8	12.6	17.8	11.1	19.2	14.5	18.1	10.3	n.s.	ANOVA
Nozue (24)	g/day	Males	31.4	14.8	30.2	13.2	N/A	N/A	28.6	12.2	30.1	-	-	Paired t tests were conducted for each two seasons
Nozue (24)	g/day	Females	25.2	11.9	23.9	10.8	N/A	N/A	22.9	10.0	24.0	-	-	Paired t tests were conducted for each two seasons
Saturated fatty acids														
Tokudome (3)	g/day	Females	15.3	6.0	15.1	6.6	16.4	6.9	15.6	6.6	15.6	-	<0.01	ANOVA
Owaki (4)	g/day	Males	11.7	5.6	12.3	5.7	11.1	5.1	11.6	5.8	11.7	4.7	n.s.	ANOVA
Owaki (4)	g/day	Females	11.2	5.0	11.5	6.1	11.0	5.2	11.9	6.6	11.4	4.7	n.s.	ANOVA
Amano (29)	g/1000 kcal	Females	9.7	2.6	9.0	2.0	9.8	2.5	9.4	2.2	9.6	-	n.s.	ANOVA
Akimoto (32)	g/1000 kcal	Males and Females	9.2	2.1	9.2	2.1	9.2	2.1	9.2	2.5	-	-	0.92	Mixed effect model
Monounsaturated fatty acids														
Tokudome (3)	g/day	Females	19.1	7.6	18.7	7.7	19.7	7.6	18.9	7.6	19.1	-	<0.05	ANOVA
Owaki (4)	g/day	Males	16.0	7.9	18.1	8.4	14.3	6.4	14.6	7.1	15.7	6.2	<0.01	ANOVA
Owaki (4)	g/day	Females	14.2	6.0	15.8	7.9	13.2	5.7	14.3	7.5	14.4	5.6	<0.05	ANOVA
Amano (29)	g/1000 kcal	Females	12.4	2.9	11.5	2.5	11.7	2.7	12.0	2.6	12.0	-	n.s.	ANOVA
Akimoto (32)	g/1000 kcal	Males and Females	12.1	2.9	11.7	2.5	12.1	2.5	11.7	2.5	-	-	0.37	Mixed effect model
Polyunsaturated fatty acids														
Tokudome (3)	g/day	Females	13.1	5.1	13.0	5.2	13.8	5.4	12.7	4.9	13.2	-	<0.01	ANOVA
Owaki (4)	g/day	Males	13.1	6.3	14.4	6.0	11.2	4.0	11.0	6.1	12.4	4.9	<0.01	ANOVA
Owaki (4)	g/day	Females	11.8	4.7	12.3	5.5	9.9	3.8	11.0	5.5	11.3	4.4	<0.01	ANOVA
Amano (29)	g/1000 kcal	Females	7.5	1.9	7.0	2.0	7.0	1.9	6.8	1.6	7.1	-	<0.05	ANOVA
Akimoto (32)	g/1000 kcal	Males and Females	7.1	5.4	6.7	5.4	7.1	5.4	7.1	5.4	-	-	0.09	Mixed effect model
n-6 Polyunsaturated fatty acids														
Tokudome (3)	g/day	Females	10.7	4.4	10.6	4.5	11.0	4.8	10.2	4.1	10.6	-	<0.01	ANOVA

eTable 2. *Cont.*

	Unit	Population	Season										P-value	P-value detail
			Spring		Summer		Fall		Winter		ALL			
			mean	SD	mean	SD	mean	SD	mean	SD	mean	SD		
n-3 Polyunsaturated fatty acids														
Tokudome (3)	g/day	Females	2.4	1.3	2.4	1.4	2.7	1.4	2.5	1.4	2.5	-	<0.001	ANOVA
Cholesterol														
Sasaki (2)	mg/day	Males	426	117	449	137	451	118	429	118	439	-	0.47	Two-way ANOVA adjusted for area difference
Sasaki (2)	mg/day	Females	360	108	379	121	376	105	367	109	371	-	0.69	Two-way ANOVA adjusted for area difference
Tokudome (3)	mg/day	Females	372	191	354	206	379	179	357	192	366	-	n.s.	ANOVA
Owaki (4)	mg/day	Males	404	258	363	287	331	207	338	261	359	154	n.s.	ANOVA
Owaki (4)	mg/day	Females	324	192	304	217	271	180	276	187	294	176	n.s.	ANOVA
Nozue (24)	mg/day	Males	403	186	346	153	N/A	N/A	373	163	374	-	-	Paired t tests were conducted for each two seasons
Nozue (24)	mg/day	Females	343	143	298	119	N/A	N/A	316	146	319	-	-	Paired t tests were conducted for each two seasons
Amano (29)	mg/1000 kcal	Females	230	70	218	71	215	80	205	86	217	-	n.s.	ANOVA
Akimoto (32)	mg/1000 kcal	Males and Females	158	6	159	6	163	6	147	6	-	-	0.13	Mixed effect model
Carbohydrate														
Sasaki (2)	g/day	Males	341	82	346	99	351	81	332	73	343	-	0.065	Two-way ANOVA adjusted for area difference
Sasaki (2)	g/day	Females	273	65	271	65	281	60	266	51	273	-	0.041	Two-way ANOVA adjusted for area difference
Tokudome (3)	g/day	Females	243	53	240	53	246	52	246	52	244	-	n.s.	ANOVA
Owaki (4)	g/day	Males	305	77	305	80	293	84	302	84	301	60	n.s.	ANOVA
Owaki (4)	g/day	Females	258	51	252	59	252	56	254	62	254	50	n.s.	ANOVA
Nozue (24)	g/day	Males	340	77	340	75	N/A	N/A	337	72	339	-	-	Paired t tests were conducted for each two seasons
Nozue (24)	g/day	Females	280	68	284	50	N/A	N/A	291	67	285	-	-	Paired t tests were conducted for each two seasons
Amano (29)	g/1000 kcal	Females	129	14	134	14	132	14	132	14	131	-	<0.05	ANOVA
Minari (31)	%E	Females	57.2	1.0	57.8	2.1	60.7	2.1	55.0	4.8	58	-	<0.05	Steel-Dwass's multiple comparison test
Akimoto (32)	g/1000 kcal	Males and Females	131	16	134	15	134	16	133	15	-	-	0.55	Mixed effect model
Total fiber														
Tokudome (3)	g/day	Females	15.1	5.4	14.7	5.3	16.4	5.4	16.5	5.5	15.7	-	<0.001	ANOVA
Owaki (4)	g/day	Males	13.5	4.5	12.1	4.2	12.0	4.0	11.2	4.1	12.2	3.8	<0.01	ANOVA
Owaki (4)	g/day	Females	13.1	5.0	11.6	4.2	11.6	3.9	12	5.5	12.1	3.8	<0.05	ANOVA
Nozue (24)	g/day	Males	17.0	5.0	17.8	5.5	N/A	N/A	18.4	5.4	17.7	-	-	Paired t tests were conducted for each two seasons
Nozue (24)	g/day	Females	16.4	6.0	17.6	4.6	N/A	N/A	19.1	5.1	17.7	-	-	Paired t tests were conducted for each two seasons
Amano (29)	g/1000 kcal	Females	6.4	1.8	6.1	1.5	6.8	1.9	6.3	2.0	6.5	-	n.s.	ANOVA
Minari (31)	g/1000 kcal	Females	13.4	1.6	12.8	1.6	13.9	1.1	16.8	4.2	14.2	-	<0.05	Steel-Dwass's multiple comparison test
Akimoto (32)	g/1000 kcal	Males and Females	7.1	5.9	6.7	5.9	7.5	5.9	7.1	5.9	-	-	0.33	Mixed effect model
Soluble dietary fiber														
Tokudome (3)	g/day	Females	2.7	1.4	3.0	1.5	3.2	1.5	3.3	1.1	3.1	-	<0.001	ANOVA
Owaki (4)	g/day	Males	2.4	0.9	2.0	1.0	2.2	1.0	2.1	0.9	2.2	0.9	n.s.	ANOVA
Owaki (4)	g/day	Females	2.4	1.1	2.0	1.0	2.2	0.9	2.2	1.1	2.2	0.8	<0.05	ANOVA
Nozue (24)	g/day	Males	3.8	1.3	4.3	1.5	N/A	N/A	4.2	1.3	4.1	-	-	Paired t tests were conducted for each two seasons
Nozue (24)	g/day	Females	3.7	1.5	4.2	1.3	N/A	N/A	4.5	1.4	4.1	-	-	Paired t tests were conducted for each two seasons
Amano (29)	g/1000 kcal	Females	1.6	0.8	1.4	0.4	1.6	0.6	1.5	0.5	1.5	-	n.s.	ANOVA

eTable 2. *Cont.*

	Unit	Population	Season										P-value	P-value detail
			Spring		Summer		Fall		Winter		ALL			
			mean	SD	mean	SD	mean	SD	mean	SD	mean	SD		
Insoluble dietary fiber														
Tokudome (3)	g/day	Females	11.7	4	11.1	3.9	12.5	5.4	12.5	4.2	12.0	-	<0.001	ANOVA
Owaki (4)	g/day	Males	10.8	3.7	9.4	3.2	9.3	2.9	8.9	3.1	9.6	3.1	<0.01	ANOVA
Owaki (4)	g/day	Females	10.2	3.5	8.7	3	9	3.1	9.3	4.1	9.3	2.8	<0.05	ANOVA
Nozue (24)	g/day	Males	13.2	3.8	13.5	4.1	N/A	N/A	14.2	4.2	13.6	-	-	Paired t tests were conducted for each two seasons
Nozue (24)	g/day	Females	12.7	4.6	13.3	3.4	N/A	N/A	14.6	3.9	13.5	-	-	Paired t tests were conducted for each two seasons
Amano (29)	g/1000 kcal	Females	4.5	1.2	4.3	1.1	4.7	1.3	4.4	1.6	4.6	-	n.s.	ANOVA
Vitamin A														
Tokudome (3)	µgRE/day	Females	715	677	707	642	824	807	779	627	756	-	<0.01	ANOVA
Owaki (4)	µgRE/day	Males	707	769	649	730	807	1160	562	366	681	494	n.s.	ANOVA
Owaki (4)	µgRE/day	Females	685	685	701	727	835	1433	663	757	721	526	n.s.	ANOVA
Nozue (24)	µgRE/day	Males	951	383	1031	677	N/A	N/A	1159	884	1047	-	-	Paired t tests were conducted for each two seasons
Nozue (24)	µgRE/day	Females	918	479	953	502	N/A	N/A	1253	837	1041	-	-	Paired t tests were conducted for each two seasons
Amano (29)	µgRE/1000 kcal	Females	449	278	382	191	463	275	390	183	427	-	n.s.	ANOVA
Minari (31)	µgRE/day	Females	590	94	545	162	534	83	681	471	588	-	<0.05	Steel-Dwass's multiple comparison test
Vitamin D														
Tokudome (3)	µg/day	Females	7.4	8.6	6.9	8.6	7.8	9.3	7.4	9.4	7.4	-	n.s.	ANOVA
Owaki (4)	IU/day	Males	122	197.9	149	252.1	118	155.7	96	175.1	121	134	n.s.	ANOVA
Owaki (4)	IU/day	Females	106	201.9	104	126	95	108.7	72	135.2	93	115	n.s.	ANOVA
Nozue (24)	µg/day	Males	9.6	5.8	12.3	10.0	N/A	N/A	9.9	5.6	10.6	-	-	Paired t tests were conducted for each two seasons
Nozue (24)	µg/day	Females	7.2	3.7	10.3	7.1	N/A	N/A	8.8	6.1	8.8	-	-	Paired t tests were conducted for each two seasons
Amano (29)	µg/1000 kcal	Females	4.0	3.0	4.0	3.0	4.0	3.0	3.0	2.0	3.9	-	n.s.	ANOVA
Vitamin E														
Tokudome (3)	mg/day	Females	8.7	3.1	9.0	3.2	9.2	3.4	8.6	3.1	8.9	-	<0.001	ANOVA
Owaki (4)	mg/day	Males	8.2	3.7	9.1	3.9	7.5	3.00	6.7	3.1	7.9	2.8	<0.01	ANOVA
Owaki (4)	mg/day	Females	7.0	2.6	8.0	3.3	6.9	2.9	6.9	2.9	7.2	2.6	<0.05	ANOVA
Nozue (24)	mg/day	Males	12.1	11.6	10.9	3.7	N/A	N/A	9.5	3.3	10.8	-	-	Paired t tests were conducted for each two seasons
Nozue (24)	mg/day	Females	10.4	12.3	9.6	3.3	N/A	N/A	9.6	3.6	9.9	-	-	Paired t tests were conducted for each two seasons
Amano (29)	mg/1000 kcal	Females	4.7	1.0	4.5	1.2	4.5	1.0	4.3	1.1	4.6	-	n.s.	ANOVA
Vitamin K														
Nozue (24)	µg/day	Males	361	172	310	158	N/A	N/A	375	172	348.7	-	-	Paired t tests were conducted for each two seasons
Nozue (24)	µg/day	Females	317	156	280	119	N/A	N/A	362	194	319.7	-	-	Paired t tests were conducted for each two seasons
Amano (29)	µg/1000 kcal	Females	116	69	100	62	139	106	109	75	116	-	<0.01	ANOVA

eTable 2. *Cont.*

	Unit	Population	Season										P-value	P-value detail
			Spring		Summer		Fall		Winter		ALL			
			mean	SD	mean	SD	mean	SD	mean	SD	mean	SD		
Thiamine														
Sasaki (2)	mg/day	Males	1.39	0.56	1.34	0.37	1.32	0.26	1.33	0.27	1.35	-	0.394	Two-way ANOVA adjusted for area difference
Sasaki (2)	mg/day	Females	1.16	0.34	1.14	0.24	1.12	0.23	1.15	0.29	1.14	-	0.627	Two-way ANOVA adjusted for area difference
Owaki (4)	mg/day	Males	0.78	0.25	0.80	0.29	0.73	0.31	0.76	0.28	0.77	0.20	n.s.	ANOVA
Owaki (4)	mg/day	Females	0.70	0.19	0.72	0.24	0.64	0.22	0.74	0.53	0.70	0.22	n.s.	ANOVA
Nozue (24)	mg/day	Males	1.18	0.9	1.19	0.7	N/A	N/A	1.15	0.8	1.17	-	-	Paired t tests were conducted for each two seasons
Nozue (24)	mg/day	Females	1.03	0.9	0.93	0.3	N/A	N/A	0.89	0.3	0.95	-	-	Paired t tests were conducted for each two seasons
Amano (29)	mg/1000 kcal	Females	0.49	0.11	0.46	0.10	0.48	0.11	0.49	0.12	0.48	-	n.s.	ANOVA
Minari (31)	mg/day	Females	0.86	0.10	0.80	0.05	0.81	0.11	1.05	0.21	0.88	-	<0.05	Steel-Dwass's multiple comparison test
Riboflavin														
Sasaki (2)	mg/day	Males	1.62	0.37	1.64	0.49	1.63	0.45	1.66	0.40	1.64	-	0.822	Two-way ANOVA adjusted for area difference
Sasaki (2)	mg/day	Females	1.43	0.30	1.43	0.31	1.41	0.30	1.47	0.34	1.44	-	0.261	Two-way ANOVA adjusted for area difference
Owaki (4)	mg/day	Males	1.07	0.34	1.07	0.42	1.04	0.41	0.95	0.31	1.03	0.28	n.s.	ANOVA
Owaki (4)	mg/day	Females	1.03	0.30	0.99	0.36	1.01	0.44	0.92	0.36	0.99	0.31	n.s.	ANOVA
Nozue (24)	mg/day	Males	1.59	1.0	1.54	0.8	N/A	N/A	1.59	0.9	1.57	-	-	Paired t tests were conducted for each two seasons
Nozue (24)	mg/day	Females	1.43	1.1	1.18	0.4	N/A	N/A	1.29	0.5	1.30	-	-	Paired t tests were conducted for each two seasons
Amano (29)	mg/1000 kcal	Females	0.63	0.30	0.59	0.13	0.65	0.17	0.59	0.14	0.62	-	<0.05	ANOVA
Minari (31)	mg/day	Females	1.13	0.16	1.14	0.11	0.92	0.11	1.43	0.42	1.16	-	<0.05	Steel-Dwass's multiple comparison test
Niacin														
Sasaki (2)	mg/day	Males	22.5	4.5	22.7	5.1	21.6	4.6	22.2	5.0	22.3	-	0.289	Two-way ANOVA adjusted for area difference
Sasaki (2)	mg/day	Females	17.5	4.3	17.2	3.6	17.0	3.2	17.1	4.0	17.2	-	0.729	Two-way ANOVA adjusted for area difference
Nozue (24)	mg/day	Males	19.9	5.9	20.6	7.2	N/A	N/A	19.7	6.8	20.1	-	-	Paired t tests were conducted for each two seasons
Nozue (24)	mg/day	Females	15.8	5.1	15.8	5.3	N/A	N/A	16.1	5.2	15.9	-	-	Paired t tests were conducted for each two seasons
Amano (29)	mg/1000 kcal	Females	8.2	2.3	8.0	2.7	8.0	2.4	8.4	2.2	8.2	-	n.s.	ANOVA
Vitamin B_6														
Nozue (24)	mg/day	Males	1.71	1.1	1.73	0.9	N/A	N/A	1.80	1.2	1.75	-	-	Paired t tests were conducted for each two seasons
Nozue (24)	mg/day	Females	1.48	1.2	1.30	0.3	N/A	N/A	1.43	0.7	1.40	-	-	Paired t tests were conducted for each two seasons
Amano (29)	mg/1000 kcal	Females	0.60	0.10	0.60	0.10	0.60	0.10	0.60	0.10	0.60	-	n.s.	ANOVA
Vitamin B_{12}														
Nozue (24)	mg/day	Males	11.8	7.7	9.7	7.3	N/A	N/A	10.6	7.0	10.7	-	-	Paired t tests were conducted for each two seasons
Nozue (24)	mg/day	Females	9.0	6.8	7.5	6.1	N/A	N/A	8.6	7.5	8.4	-	-	Paired t tests were conducted for each two seasons
Amano (29)	µg/1000 kcal	Females	3.0	1.8	2.8	1.7	3.1	2.3	2.7	1.8	2.9	-	n.s.	ANOVA
Folate														
Nozue (24)	µg/day	Males	378	118	380	124	N/A	N/A	412	137	390	-	-	Paired t tests were conducted for each two seasons
Nozue (24)	µg/day	Females	353	108	337	93	N/A	N/A	407	136	366	-	-	Paired t tests were conducted for each two seasons
Amano (29)	µg/1000 kcal	Females	154	49	136	40	168	70	140	37	150	-	<0.01	ANOVA
Ishiwaki (30)	µg/day	Males	444	155	430	252	420	172	427	166	430	135	>0.05	ANOVA
Ishiwaki (30)	µg/day	Females	408	136	406	335	406	160	403	144	406	141	>0.05	ANOVA

eTable 2. *Cont.*

	Unit	Population	Season										P-value	P-value detail
			Spring		Summer		Fall		Winter		ALL			
			mean	SD	mean	SD	mean	SD	mean	SD	mean	SD		
Pantothenic acid														
Nozue (24)	mg/day	Males	6.59	1.9	6.99	2.0	N/A	N/A	6.66	2.0	6.75	-	-	Paired t tests were conducted for each two seasons
Nozue (24)	mg/day	Females	5.80	1.8	5.90	1.4	N/A	N/A	5.97	1.8	5.89	-	-	Paired t tests were conducted for each two seasons
Amano (29)	mg/1000 kcal	Females	3.19	0.55	3.06	0.43	3.33	0.68	3.05	0.48	3.16	-	<0.01	ANOVA
Vitamin C														
Sasaki (2)	mg/day	Males	113	41	127	76	154	60	130	41	131	-	<0.001	Two-way ANOVA adjusted for area difference
Sasaki (2)	mg/day	Females	120	48	131	57	163	63	145	51	140	-	<0.001	Two-way ANOVA adjusted for area difference
Tokudome (3)	mg/day	Females	136	72	128	78	160	82	154	74	145	-	<0.001	ANOVA
Owaki (4)	mg/day	Males	74	43	56	31	48	31	57	35	59	30	<0.01	ANOVA
Owaki (4)	mg/day	Females	80	37	58	32	53	29	59	29	63	30	<0.01	ANOVA
Nozue (24)	mg/day	Males	304	637	102	36	N/A	N/A	292	559	233	-	-	Paired t tests were conducted for each two seasons
Nozue (24)	mg/day	Females	561	1873	105	35	N/A	N/A	585	2176	417	-	-	Paired t tests were conducted for each two seasons
Amano (29)	mg/1000 kcal	Females	46	21	39	20	48	23	36	17	42	-	<0.05	ANOVA
Ishiwaki (30)	mg/day	Males	120	53	124	84	145	81	125	61	128	49	<0.001	ANOVA
Ishiwaki (30)	mg/day	Females	132	77	123	85	158	84	137	77	138	58	<0.001	
Minari (31)	mg/day	Females	98	18	90	8	99	8	145	54	108	-	<0.05	Steel-Dwass's multiple comparison test
Sodium and Salt														
Sasaki (2)	mg/day	Males	5607	1264	5881	1357	5813	1231	5851	1472	5788	-	0.139	Two-way ANOVA adjusted for area difference
Sasaki (2)	mg/day	Females	4779	1147	5103	1091	5033	1168	5226	1299	5035	-	0.003	Two-way ANOVA adjusted for area difference
Owaki (4)	salt eq g/day	Males	13.7	4.2	15.1	4.8	12.8	3.5	13.6	3.5	13.8	3.2	<0.05	ANOVA
Owaki (4)	salt eq g/day	Females	13.2	4.4	13.5	3.7	12.8	3.2	12	3.4	12.9	3.3	<0.05	ANOVA
Nozue (24)	salt eq g/day	Males	14.2	4.7	14.9	6.6	N/A	N/A	15.0	4.2	14.7	-	-	Paired t tests were conducted for each two seasons
Nozue (24)	salt eq g/day	Females	12.2	3.2	12.5	3.5	N/A	N/A	13.4	3.5	12.7	-	-	Paired t tests were conducted for each two seasons
Amano (29)	mg/1000 kcal	Females	1943	430	2006	405	1907	495	1910	419	1942	-	n.s.	ANOVA
Ishiwaki (30)	salt eq g/day	Males	14.5	4.6	14.5	4.4	13.9	4.2	13.6	3.9	14.1	3.3	<0.01	ANOVA
Ishiwaki (30)	salt eq g/day	Females	12.3	3.8	12.4	4.3	12.1	3.7	11.8	3.4	12.2	3.0	<0.05	ANOVA
Minari (31)	salt eq g/day	Females	11.4	2.6	9.1	1.1	8.7	0.5	10.9	2.1	10.0	-	<0.05	Steel-Dwass's multiple comparison test
Potassium														
Sasaki (2)	mg/day	Males	3281	657	3406	787	3343	718	3380	712	3353	-	0.271	Two-way ANOVA adjusted for area difference
Sasaki (2)	mg/day	Females	2967	651	3114	696	3032	645	3119	772	3058	-	0.05	Two-way ANOVA adjusted for area difference
Tokudome (3)	mg/day	Females	2810	795	2817	775	3056	763	2969	780	2913	-	<0.001	ANOVA
Owaki (4)	mg/day	Males	3081	836	3234	983	2866	968	2775	713	2989	647	<0.01	ANOVA
Owaki (4)	mg/day	Females	3053	834	3067	921	2761	795	2743	786	2906	717	<0.01	ANOVA
Nozue (24)	mg/day	Males	2846	725	3044	826	N/A	N/A	2985	777	2958	-	-	Paired t tests were conducted for each two seasons
Nozue (24)	mg/day	Females	2667	812	2825	703	N/A	N/A	2865	723	2786	-	-	Paired t tests were conducted for each two seasons
Amano (29)	mg/1000 kcal	Females	1168	251	1135	272	1246	286	1138	222	1172	-	<0.05	ANOVA

eTable 2. *Cont.*

			Season											
			Spring		Summer		Fall		Winter		ALL			
	Unit	Population	mean	SD	mean	SD	mean	SD	mean	SD	mean	SD	P-value	P-value detail
Calcium														
Sasaki (2)	mg/day	Males	646	174	667	215	691	209	692	209	674	-	0.093	Two-way ANOVA adjusted for area difference
Sasaki (2)	mg/day	Females	616	159	623	174	632	181	662	179	633	-	0.041	Two-way ANOVA adjusted for area difference
Tokudome (3)	mg/day	Females	602	239	623	245	662	250	642	257	632	-	<0.001	ANOVA
Owaki (4)	mg/day	Males	599	245	607	282	583	342	532	221	580	205	n.s.	ANOVA
Owaki (4)	mg/day	Females	640	252	601	271	585	270	594	255	605	224	n.s.	ANOVA
Nozue (24)	mg/day	Males	667	308	593	256	N/A	N/A	640	297	633	-	-	Paired t tests were conducted for each two seasons
Nozue (24)	mg/day	Females	704	564	556	188	N/A	N/A	688	479	649	-	-	Paired t tests were conducted for each two seasons
Amano (29)	mg/1000 kcal	Females	256	87	252	93	279	114	228	78	254	-	<0.01	ANOVA
Ishiwaki (30)	mg/day	Males	661	239	634	231	643	223	654	218	648	189	>0.05	ANOVA
Ishiwaki (30)	mg/day	Females	646	236	616	231	634	224	360	230	631	188	>0.05	ANOVA
Minari (31)	mg/day	Females	474	76	459	58	448	44	602	178	496	-	<0.05	Steel-Dwass's multiple comparison test
Magnesium														
Tokudome (3)	mg/day	Females	248	74	249	73	264	72	258	73	255	-	<0.001	ANOVA
Owaki (4)	mg/day	Males	251	67	260	81	246	64	225	67	246	61	<0.05	ANOVA
Owaki (4)	mg/day	Females	220	68	219	69	204	61	198	58	210	57	<0.05	ANOVA
Nozue (24)	mg/day	Males	329	79	342	96	N/A	N/A	315	69	329	-	-	Paired t tests were conducted for each two seasons
Nozue (24)	mg/day	Females	299	84	304	70	N/A	N/A	289	69	297	-	-	Paired t tests were conducted for each two seasons
Amano (29)	mg/1000 kcal	Females	120	25	120	29	126	35	119	28	121	-	n.s.	ANOVA
Phosphorus														
Sasaki (2)	mg/day	Males	1486	261	1509	301	1506	283	1491	283	1498	-	0.749	Two-way ANOVA adjusted for area difference
Sasaki (2)	mg/day	Females	1230	225	1220	218	1214	212	1228	238	1223	-	0.882	Two-way ANOVA adjusted for area difference
Tokudome (3)	mg/day	Females	1063	262	1070	274	1096	237	1077	262	1077	-	n.s.	ANOVA
Owaki (4)	mg/day	Males	1308	385	1319	425	1250	389	1218	299	1274	256	n.s.	ANOVA
Owaki (4)	mg/day	Females	1150	276	1139	309	1076	287	1092	301	1114	257	n.s.	ANOVA
Nozue (24)	mg/day	Males	1277	332	1234	307	N/A	N/A	1215	282	1242	-	-	Paired t tests were conducted for each two seasons
Nozue (24)	mg/day	Females	1096	283	1044	249	N/A	N/A	1049	252	1063	-	-	Paired t tests were conducted for each two seasons
Amano (29)	mg/1000 kcal	Females	538	79	535	86	549	100	512	77	533	-	<0.05	ANOVA

eTable 2. *Cont.*

	Unit	Population	Season								ALL		P-value	P-value detail
			Spring		Summer		Fall		Winter					
			mean	SD	mean	SD	mean	SD	mean	SD	mean	SD		
Iron														
Sasaki (2)	mg/day	Males	13.2	2.7	13.0	3.1	13.3	2.9	14.0	3.2	13.4	-	0.004	Two-way ANOVA adjusted for area difference
Sasaki (2)	mg/day	Females	11.6	2.6	11.3	2.3	11.6	2.4	12.6	2.9	11.8	-	<0.001	Two-way ANOVA adjusted for area difference
Tokudome (3)	mg/day	Females	10.6	3.2	10.1	2.9	11.4	3.3	11.2	3.2	10.8	-	<0.001	ANOVA
Owaki (4)	mg/day	Males	12.7	3.1	12.8	3.5	11.7	3.8	11.7	3.8	12.2	2.5	<0.05	ANOVA
Owaki (4)	mg/day	Females	12.3	3.9	11.8	3.6	11.3	3.4	11.3	4.1	11.7	2.9	n.s.	ANOVA
Nozue (24)	mg/day	Males	10.2	2.7	9.8	2.7	N/A	N/A	10.0	2.4	10.0	-	-	Paired t tests were conducted for each two seasons
Nozue (24)	mg/day	Females	9.6	2.9	9.0	2.1	N/A	N/A	9.6	2.6	9.4	-	-	Paired t tests were conducted for each two seasons
Amano (29)	mg/1000 kcal	Females	4.0	0.8	4.0	0.9	4.1	1.0	4.0	1.0	4.0	-	n.s.	ANOVA
Ishiwaki (30)	mg/day	Males	10.7	3.1	10.2	4.0	10.1	2.9	10.4	2.9	10.4	2.5	>0.05	ANOVA
Ishiwaki (30)	mg/day	Females	9.6	2.8	9.5	5.3	9.5	3.0	9.7	3.3	9.6	2.8	>0.05	ANOVA
Minari (31)	mg/day	Females	8.8	1.0	8.2	1.1	8.3	0.1	10.2	2.6	8.9	-	<0.05	Steel-Dwass's multiple comparison test
Zinc														
Tokudome (3)	mg/day	Females	7.8	2.1	7.8	2.2	8.7	5.1	9.5	7.5	8.5	-	<0.001	ANOVA
Owaki (4)	mg/day	Males	7.5	2.5	7.7	2.8	7.3	2.0	6.8	2.1	7.3	2.1	n.s.	ANOVA
Owaki (4)	mg/day	Females	6.3	1.6	6.2	1.8	6.2	2.1	5.8	1.7	6.1	1.6	n.s.	ANOVA
Nozue (24)	mg/day	Males	10.4	2.4	10.0	2.4	N/A	N/A	10.1	2.2	10.2	-	-	Paired t tests were conducted for each two seasons
Nozue (24)	mg/day	Females	8.9	2.4	8.4	1.7	N/A	N/A	8.7	2.5	8.7	-	-	Paired t tests were conducted for each two seasons
Amano (29)	mg/1000 kcal	Females	4.5	0.6	4.4	0.7	4.4	0.7	4.4	0.8	4.4	-	n.s.	ANOVA
Copper														
Tokudome (3)	mg/day	Females	1.2	0.4	1.1	0.3	1.3	0.6	1.3	0.7	1.2	-	<0.001	ANOVA
Owaki (4)	mg/day	Males	1.1	0.4	1.2	0.5	1.2	0.7	1.1	0.5	1.2	0.4	n.s.	ANOVA
Owaki (4)	mg/day	Females	0.9	0.3	1.0	0.4	1.0	0.7	1.0	0.6	1.0	0.4	n.s.	ANOVA
Nozue (24)	mg/day	Males	1.6	0.4	1.6	0.4	N/A	N/A	1.6	0.4	1.6	-	-	Paired t tests were conducted for each two seasons
Nozue (24)	mg/day	Females	1.4	0.4	1.4	0.3	N/A	N/A	1.4	0.3	1.4	-	-	Paired t tests were conducted for each two seasons
Amano (29)	mg/1000 kcal	Females	0.6	0.1	0.6	0.2	0.6	0.1	0.6	0.2	0.6	-	n.s.	ANOVA

SD, Standard deviation. N/A, Not assessed. "-" means the SD was not calculated in the study. n.s. means there were no significant differences in intakes between seasons.

eTable 3. Mean intake of food groups in each season

	Unit	Population	Season										P-value	P-value detail
			Spring		Summer		Fall		Winter		ALL			
			mean	SD	mean	SD	mean	SD	mean	SD	mean	SD		
Grains														
Sasaki (2)	g/day	Males	331	97	332	113	338	94	324	92	331	-	0.469	Two-way ANOVA adjusted for area difference
Sasaki (2)	g/day	Females	234	59	228	66	233	66	220	50	229	-	0.157	Two-way ANOVA adjusted for area difference
Nozue (24)	g/day	Males	606	182	592	147	N/A	N/A	569	124	589	-	-	Paired t test
Nozue (24)	g/day	Females	441	114	441	86	N/A	N/A	431	90	438	-	-	Paired t test
Miyai (25)	g/1000 kcal	Normal weight Females	232	82	236	56	202	74	232	72	223	54	0.104	Paired friedman test
Miyai (25)	g/1000 kcal	Overweight Females	288	51	286	32	257	61	272	26	273	22	0.615	Paired friedman test
Potatoes														
Sasaki (2)	g/day	Males	37.0	27.0	39.0	29.0	63.0	35.0	55.0	34.0	48.5	-	<0.001	Two-way ANOVA adjusted for area difference
Sasaki (2)	g/day	Females	36.0	25.0	39.0	24.0	61.0	37.0	55.0	32.0	47.8	-	<0.001	Two-way ANOVA adjusted for area difference
Nozue (24)	g/day	Males	78.8	46.8	69.9	55.0	N/A	N/A	85.3	58.0	78.0	-	-	Paired t test
Nozue (24)	g/day	Females	73.5	47.6	48.7	43.0	N/A	N/A	88.1	48.7	70.1	-	-	Paired t test
Tatsumi (26)	g/day	Males and Females	31.2	23.9	28.2	23.4	40.7	22.5	39.4	22.5	34.9	-	<0.001	A general linear model for repeated measures was used to estimate area-adjusted means and 95% CI
Sugar and sweeteners														
Sasaki (2)	g/day	Males	8.0	6.0	8.0	7.0	8.0	7.0	10.0	8.0	8.5	-	0.158	Two-way ANOVA adjusted for area difference
Sasaki (2)	g/day	Females	9.0	7.0	8.0	6.0	8.0	5.0	9.0	7.0	8.5	-	0.102	Two-way ANOVA adjusted for area difference
Nozue (24)	g/day	Males	9.5	6.3	9.9	9.4	N/A	N/A	7.6	4.4	9.0	-	-	Paired t test
Nozue (24)	g/day	Females	8.9	6.6	8.6	7.9	N/A	N/A	7.4	4.5	8.3	-	-	Paired t test
Beans														
Sasaki (2)	g/day	Males	94.0	47.0	85.0	39.0	91.0	44.0	94.0	44.0	91.0	-	0.306	Two-way ANOVA adjusted for area difference
Sasaki (2)	g/day	Females	82.0	38.0	73.0	33.0	80.0	33.0	85.0	38.0	80.0	-	0.028	Two-way ANOVA adjusted for area difference
Nozue (24)	g/day	Males	71.3	50.5	71.9	54.8	N/A	N/A	64.0	45.8	69.1	-	-	Paired t test
Nozue (24)	g/day	Females	84.1	62.2	82.5	57.7	N/A	N/A	71.3	53.9	79.3	-	-	Paired t test
Tatsumi (26)	g/day	Males and Females	65.0	28.1	63.8	29.1	63.9	28.1	73.1	29.1	66.5	-	<0.001	A general linear model for repeated measures was used to estimate area-adjusted means and 95% CI
Nuts and seeds														
Sasaki (2)	g/day	Males	2.0	3.0	2.0	3.0	4.0	9.0	3.0	6.0	2.8	-	0.053	Two-way ANOVA adjusted for area difference
Sasaki (2)	g/day	Females	2.0	4.0	2.0	2.0	6.0	18.0	3.0	5.0	3.3	-	0.069	Two-way ANOVA adjusted for area difference
Nozue (24)	g/day	Males	2.1	3.0	2.4	8.3	N/A	N/A	1.5	3.2	2.0	-	-	Paired t test
Nozue (24)	g/day	Females	2.6	3.4	2.4	4.3	N/A	N/A	1.9	3.2	2.3	-	-	Paired t test
Vegetables														
Sasaki (2)	g/day	Males	310	92	388	157	299	98	302	97	325	-	<0.001	Two-way ANOVA adjusted for area difference
Sasaki (2)	g/day	Females	298	94	391	134	278	98	294	110	315	-	<0.001	Two-way ANOVA adjusted for area difference
Nozue (24)	g/day	Males	316	103	463	182	N/A	N/A	386	125	388	-	-	Paired t test
Nozue (24)	g/day	Females	290	99	457	154	N/A	N/A	378	121	375	-	-	Paired t test
Tatsumi (26)	g/day	Males and Females	253	72	272	71	239	72	270	71	259	-	<0.001	A general linear model for repeated measures was used to estimate area-adjusted means and 95% CI

eTable 3. *Cont.*

	Unit	Population	Season										P-value	P-value detail
			Spring		Summer		Fall		Winter		ALL			
			mean	SD	mean	SD	mean	SD	mean	SD	mean	SD		
Fruits														
Sasaki (2)	g/day	Males	109	95	166	146	167	129	119	84	140	-	<0.001	Two-way ANOVA adjusted for area difference
Sasaki (2)	g/day	Females	141	96	193	133	209	100	160	93	176	-	<0.001	Two-way ANOVA adjusted for area difference
Nozue (24)	g/day	Males	91	83	125	158	N/A	N/A	142	177	120	-	-	Paired t test
Nozue (24)	g/day	Females	121	106	206	154	N/A	N/A	199	182	175	-	-	Paired t test
Tatsumi (26)	g/day	Males and Females	110	66	147	65	166	66	116	65	135	-	<0.001	A general linear model for repeated measures was used to estimate area-adjusted means and 95% CI
Mushrooms														
Sasaki (2)	g/day	Males	6.0	7.0	8.0	10.0	12.0	12.0	12.0	10.0	9.5	-	<0.001	Two-way ANOVA adjusted for area difference
Sasaki (2)	g/day	Females	6.0	6.0	8.0	8.0	13.0	12.0	10.0	9.0	9.3	-	<0.001	Two-way ANOVA adjusted for area difference
Nozue (24)	g/day	Males	22.0	19.7	12.6	13.4	N/A	N/A	20.2	16.2	18.3	-	-	Paired t test
Nozue (24)	g/day	Females	19.9	18.8	11.2	11.5	N/A	N/A	17.8	14.4	16.3	-	-	Paired t test
Seaweeds														
Sasaki (2)	g/day	Males	8.0	8.0	11.0	17.0	6.0	8.0	6.0	5.0	7.8	-	0.031	Two-way ANOVA adjusted for area difference
Sasaki (2)	g/day	Females	8.0	11.0	10.0	16.0	6.0	7.0	6.0	5.0	7.5	-	0.069	Two-way ANOVA adjusted for area difference
Nozue (24)	g/day	Males	18.3	20.3	21.8	23.1	N/A	N/A	11.2	12.6	17.1	-	-	Paired t test
Nozue (24)	g/day	Females	14.9	16.8	24.1	24.4	N/A	N/A	11.5	15.9	16.8	-	-	Paired t test
Fish and shellfish														
Sasaki (2)	g/day	Males	143	52	143	50	154	48	151	59	148	-	0.190	Two-way ANOVA adjusted for area difference
Sasaki (2)	g/day	Females	111	41	113	40	123	41	116	41	116	-	0.068	Two-way ANOVA adjusted for area difference
Nozue (24)	g/day	Males	154	68	124	74	N/A	N/A	129	59	135	-	-	Paired t test
Nozue (24)	g/day	Females	112	58	93	56	N/A	N/A	109	55	105	-	-	Paired t test
Meats														
Sasaki (2)	g/day	Males	62.0	27.0	67.0	33.0	66.0	31.0	66.0	36.0	65.3	-	0.503	Two-way ANOVA adjusted for area difference
Sasaki (2)	g/day	Females	52.0	22.0	52.0	24.0	50.0	21.0	52.0	32.0	51.5	-	0.780	Two-way ANOVA adjusted for area difference
Nozue (24)	g/day	Males	65.3	42.0	80.9	56.0	N/A	N/A	80.7	55.3	75.6	-	-	Paired t test
Nozue (24)	g/day	Females	56.7	58.8	57.2	40.0	N/A	N/A	56.8	43.0	56.9	-	-	Paired t test
Eggs														
Sasaki (2)	g/day	Males	41.0	19.0	42.0	19.0	42.0	19.0	40.0	16.0	41.3	-	0.819	Two-way ANOVA adjusted for area difference
Sasaki (2)	g/day	Females	35.0	17.0	35.0	20.0	36.0	17.0	33.0	15.0	34.8	-	0.659	Two-way ANOVA adjusted for area difference
Nozue (24)	g/day	Males	42.6	36.7	36.5	27.9	N/A	N/A	39.8	27.5	39.6	-	-	Paired t test
Nozue (24)	g/day	Females	39.1	28.7	36.4	26.1	N/A	N/A	33.8	22.5	36.4	-	-	Paired t test
Dairy														
Sasaki (2)	g/day	Males	141	125	156	145	134	133	131	106	141	-	0.114	Two-way ANOVA adjusted for area difference
Sasaki (2)	g/day	Females	166	102	178	110	140	104	140	93	156	-	<0.001	Two-way ANOVA adjusted for area difference
Nozue (24)	g/day	Males	98	159	121	175	N/A	N/A	105	158	108	-	-	Paired t test
Nozue (24)	g/day	Females	104	137	120	114	N/A	N/A	90	99	104	-	-	Paired t test

eTable 3. *Cont.*

	Unit	Population	Season								ALL		P-value	P-value detail
			Spring		Summer		Fall		Winter					
			mean	SD	mean	SD	mean	SD	mean	SD	mean	SD		
Fats and oils														
Sasaki (2)	g/day	Males	10.0	5.0	11.0	4.0	9.0	5.0	9.0	4.0	9.8	-	0.007	Two-way ANOVA adjusted for area difference
Sasaki (2)	g/day	Females	8.0	4.0	10.0	5.0	8.0	4.0	8.0	4.0	8.5	-	0.002	Two-way ANOVA adjusted for area difference
Nozue (24)	g/day	Males	11.7	7.5	14.3	9.3	N/A	N/A	9.5	6.9	11.8	-	-	Paired t test
Nozue (24)	g/day	Females	8.4	6.0	9.1	6.7	N/A	N/A	7.5	5.7	8.3	-	-	Paired t test
Miyai (25)	g/1000 kcal	Normal weight Females	5.2	3.2	8.4	5.6	8.2	4.4	6.6	3.8	7.0	2.7	0.065	Paired friedman test
Miyai (25)	g/1000 kcal	Overweight Females	6.5	2.8	6.8	3.9	6.7	3.1	5.6	2.3	6.4	1.0	0.978	Paired friedman test
Seasonings and spices														
Sasaki (2)	g/day	Males	40.0	13.0	39.0	13.0	39.0	11.0	40.0	13.0	39.5	-	0.563	Two-way ANOVA adjusted for area difference
Sasaki (2)	g/day	Females	37.0	13.0	36.0	12.0	35.0	10.0	36.0	12.0	36.0	-	0.401	Two-way ANOVA adjusted for area difference
Nozue (24)	g/day	Males	119.8	73.9	114.8	64.2	N/A	N/A	128.8	80.6	121.1	-	-	Paired t test
Nozue (24)	g/day	Females	87.1	40.1	92.3	43.0	N/A	N/A	98.4	51.3	92.6	-	-	Paired t test

SD, Standard deviation. N/A, Not assessed. "-" means the SD was not calculated in the study. SDs for potatoes, vegetables, and fruits in Tatsumi (26) were estimated from the 95% confidence intervals.

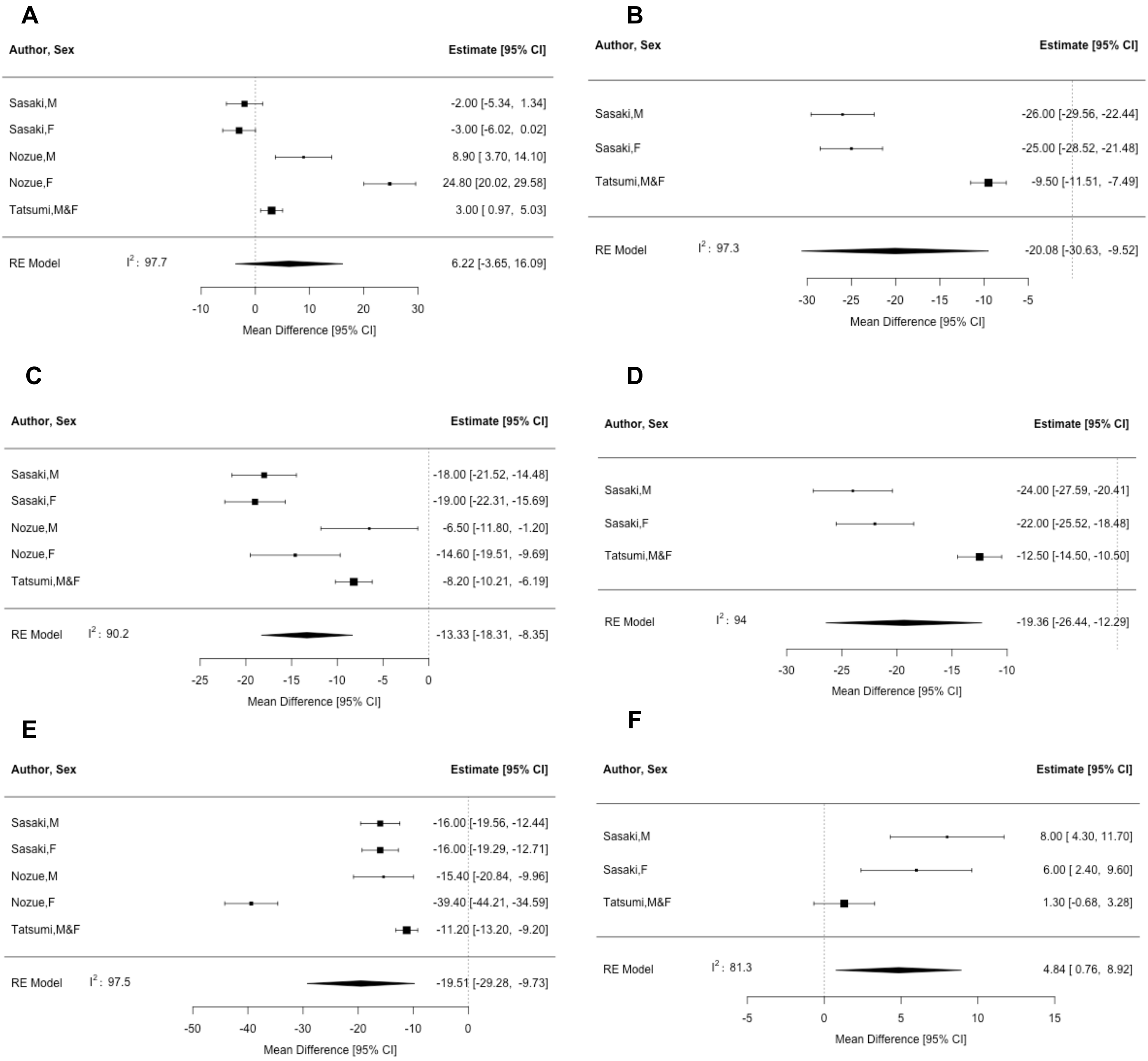

eFigure 1. Forest plot of the pooled mean differences in potato intakes. Spring vs Summer (A), Spring vs Fall (B), Spring vs Winter (C), Summer vs Fall (D), Summer vs Winter (E), Fall vs Winter (F). Mean differences were calculated by subtracting the intake in the right season from the intake in the left season. M indicates males and F indicates females.

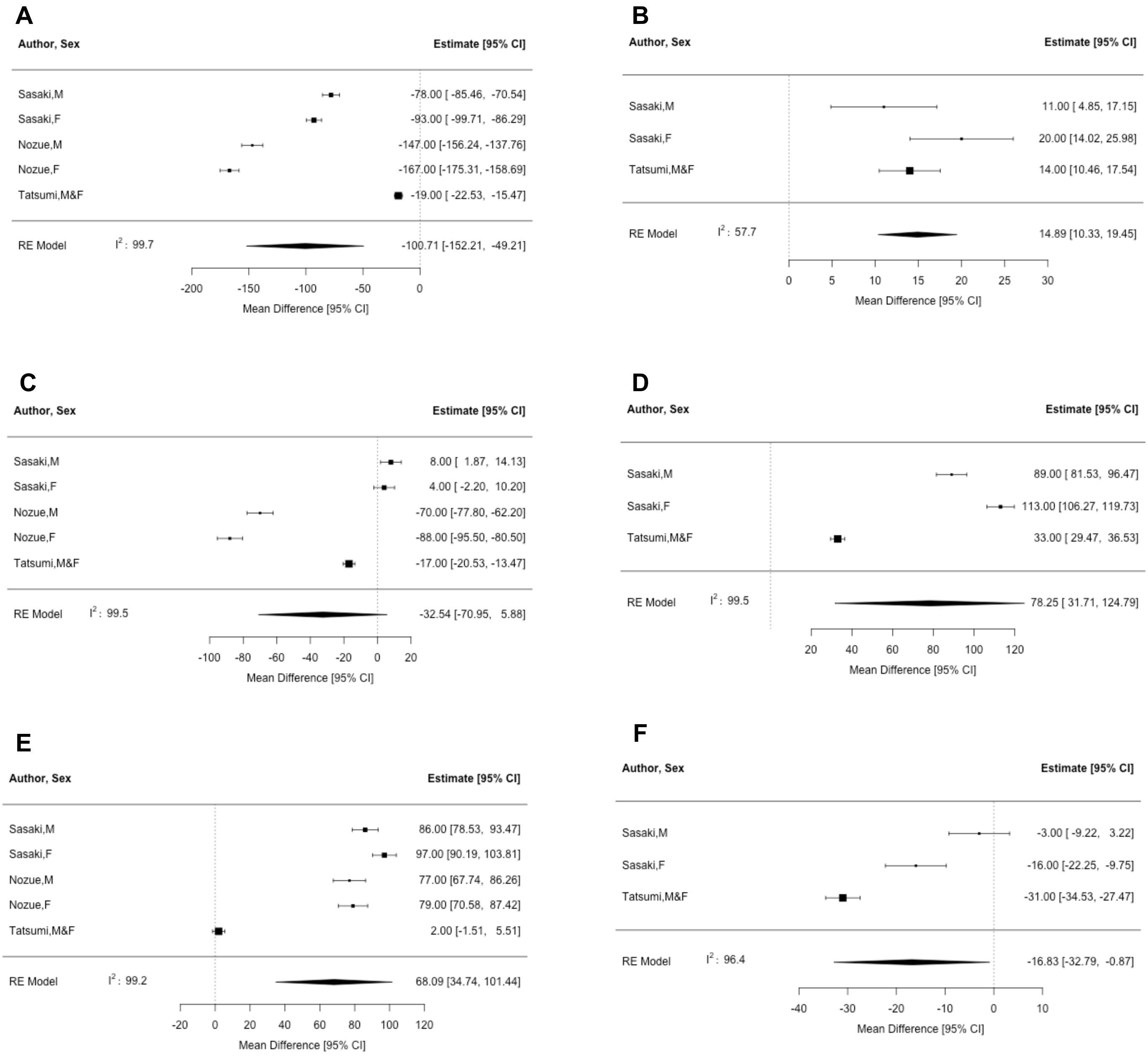

eFigure 2. Forest plot of the pooled mean differences in vegetable intakes. Spring vs Summer (A), Spring vs Fall (B), Spring vs Winter (C), Summer vs Fall (D), Summer vs Winter (E), Fall vs Winter (F). Mean differences were calculated by subtracting the intake in the right season from the intake in the left season. M indicates males and F indicates females.

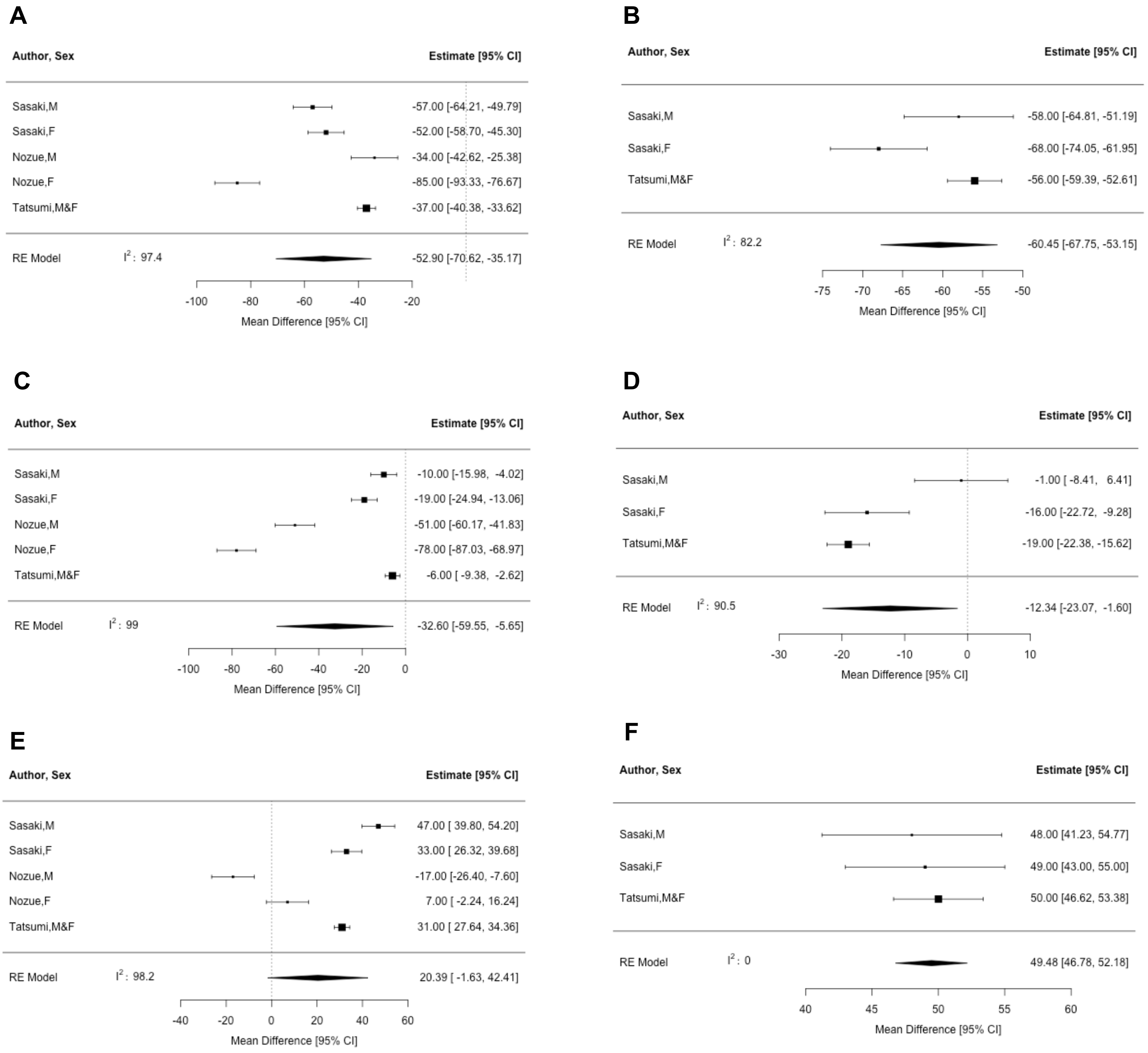

eFigure 3. Forest plot of the pooled mean differences in fruit intakes. Spring vs Summer (A), Spring vs Fall (B), Spring vs Winter (C), Summer vs Fall (D), Summer vs Winter (E), Fall vs Winter (F). Mean differences were calculated by subtracting the intake in the right season from the intake in the left season. M indicates males and F indicates females.
